# Neurological presentations of intravascular lymphoma (IVL): meta-analysis of 654 patients

**DOI:** 10.1186/s12883-015-0509-8

**Published:** 2016-01-16

**Authors:** Ekokobe Fonkem, Samantha Dayawansa, Edana Stroberg, Edwin Lok, Paul C. Bricker, Batool Kirmani, Eric T. Wong, Jason H. Huang

**Affiliations:** Department of Neurosurgery, Baylor Scott & White Health, Temple, Texas 76508 USA; Department of Neurology, Baylor Scott & White Health, Temple, Texas 76508 USA; Department of Neurology, Brain Tumor Center and Neuro-Oncology Unit, Beth Israel Deaconess Medical Center, 330 Brookline Ave, Boston, Massachusetts 02215 USA; Department of Pathology, Baylor Scott & White Health, Temple, Texas 76508 USA

**Keywords:** Intravascular lymphoma (IVL), Neurogenic manifestations

## Abstract

**Background:**

Patients with intravascular lymphoma (IVL) frequently have neurological signs and symptoms. Prompt diagnosis and treatment is therefore crucial for their survival. However, the spectrum of neurological presentations and their respective frequencies have not been adequately characterized. Our aim is to document the spectrum of clinical symptoms and their respective frequencies and to create a clinical framework for the prompt diagnosis of IVL.

**Methods:**

A comprehensive meta-analysis of 654 cases of IVL published between 1957 and 2012 was performed to provide better insight into the neurological presentations of this disease. Neurologic complications were mainly divided into central nervous system (CNS) and peripheral nervous system (PNS) presentations.

**Results:**

There were no differences in occurrences of CNS IVL based on gender or geographic locations (Asian Vs non-Asian). However, most patients with CNS IVL were younger than 70 years of age (*p* < 0.05). Our limited data do not support the treatment efficacy of methotrexate.

CNS symptoms were seen in 42 % of all cases. The most common CNS complications identified were cognitive impairment/dementia (60.9 %), paralysis (22.2 %), and seizures (13.4 %). PNS complications were seen in 9.5 % of cases. Out of these, muscle weakness (59.7 %), neurogenic bladder (37.1 %), and paresthesia (16.1 %) were the most common presentations.

**Conclusions:**

CNS complications are more common among IVL patients. Out of these, dementia and seizures outnumber stroke-like presentations.

**Electronic supplementary material:**

The online version of this article (doi:10.1186/s12883-015-0509-8) contains supplementary material, which is available to authorized users.

## Background

Intravascular lymphoma (IVL) is a rare, aggressive systemic B-cell lymphoma first described by Pfleger and Tappeiner in 1959 [1]. IVL has an estimated annual incidence of 0.5 cases per 1,000,000 population [2]. The disease is characterized by selective growth of lymphoma cells within the lumina of vessels, particularly small- and medium-sized blood vessels [3]. The unique tropism of these cells to those sites is due to the absence of CD29 and CD54 surface ligands, which may possibly limit the ability to cross the endothelium. Proliferations of large lymphoma cells within the lumen of small- to medium-sized vessels are usually observed [3]. Over time, these tumor foci may obstruct the arterial blood supply to the distal locations causing ischemia in various organs, resulting in systemic symptoms, neurological deficits, or both.

Depending on the target organ, the clinical signs and symptoms can vary. The tumor also has the ability to affect multiple organs and spread rapidly. Therefore, the clinical presentation of patients with IVL can be highly variable and unpredictable [4, 5]. Due to the rarity of this tumor and lack of sensitive diagnostic methods, it is difficult to diagnose in a timely fashion, sometimes leading to its spread.

If not diagnosed and treated early, patients with IVL survive less than a year. In more than 50 % of the cases, the diagnosis is made incidentally during autopsy [6]. However, in some cases where early diagnosis is made, remission is a possibility with the timely administration of systemic chemotherapy [6]. Therefore, proper understanding of the disease presentation will allow early diagnosis and prompt intervention. The rapid lethality of the disease in the majority of patients precludes the possibility of prospective studies. Published literature consists solely of case reports and retrospective case series. As a result, we performed a meta-analysis of the existing literature to compile and analyze the pattern of clinical presentations of IVL.

## Methods

A comprehensive literature search of intravascular lymphoma, angioendotheliomatosis, and intravascular lymphomatosis was conducted in English language electronic and print journals using PubMed, Medline, Paperchase, and Web of Science from the year 1957 to 2012. It is worth noting that in 1986 the nomenclature changed from “malignant angioendotheliomatosis” to “intravascular lymphomatosis.” Cases were not included if they were not in English or were not human studies. Also, cases were not utilized if they did not list the symptoms demonstrated by the patient upon presentation. For each case, the presentations were recorded and further classified as central nervous system (CNS), peripheral nervous system (PNS), or non-neurologic. The list of cases we utilized is attached as a supplementary file.

### Source data

Source data of this publication are presented in Additional file 1: Table S1.

### Statistics

Two-tailed Wilcoxon rank sum test was used to compare CNS, PNS, and other cases of Asian, non-Asian patients and treatment efficacy of methotrexate use.

## Results

### Patient characteristics

A total of 654 patients were included in this study (Additional file 1: Table S1). While 316 (48 %) patients presented with various non-neurologic symptoms, neurologic complications were observed in more than half of the population or 338 patients (52 %). Of those, 276 (82 %) patients presented with CNS symptoms, 62 (19 %) patients presented with PNS symptoms, and 316 (48 %) patients presented with various non-neurologic symptoms. Relative to the number of CNS patients were PNS only patient number was lesser. Due to the small population size of the PNS group and its similarity to the CNS than with other sites, these patients were combined with the CNS cohort.

The study group consisted of 137 women and 133 men (*p* = 0.43). The median age for men was 63 years (range = 17 – 86), and for women, 62 years (range = 23 – 88).

The time from symptom to death between IVL patients with age less than 70 years (*n* = 193) was 15 months (range 1 – 110) while IVL patients with age greater than or equal to 70 years (*n* = 77) was 14 months (range 0 – 63) (*P* = 0.0911). The overall survival between IVL patients with age less than 70 years was 12 months (range 0 – 96) versus IVL patients with age greater than or equal to 70 years was 10 months (range 0 – 63) (*P* = 0.1102). The time from treatment to death for IVL patients with age less than 70 years was 11 months (range 0 – 96) versus patients with age greater than or equal to 70 years was 10 months (range 0 – 63) (*P* = 0.2068). By using an unsupervised modified binary partitioning algorithm [7, 8], the age cutoff yielding the greatest time from symptom to death was age 46 years (*P* = 0.0284). The median time from symptom to death with age less than or equal to 46 was 13 months (range 3 – 54) versus 16 months (range 0 – 110). By using the same partitioning algorithm, the age cutoff yielding the greatest separation of overall survival, though it did not reach statistical significance was also 46 (*P* = 0.0786). The median overall survival with age less than or equal to 46 was 9 months (range 1 – 52) for patients with age less than or equal to 46, and 12 months (range 0 – 96) for patients with age greater than 46. Finally, the optimal age cutoff that yields the greatest separation for time from treatment to death was also 46 (*P* = 0.0583) with median time from treatment to death for patients with age less than or equal to 46 versus patients with age greater than 46 was 8 months (range 1 – 52) and 11 months (range 0 – 96), respectively.

### Methotrexate use

Median time from symptom to death for CNS methotrexate users (*n* = 6) was 15 months (range 9 – 28) versus 18 months (range 9 – 55) for other methotrexate users (*n* = 6) (*P* = 0.9357). The median overall survival for CNS methotrexate users was 14 months (range 1 – 28) versus 16 months (range 8 – 24) for other methotrexate users (*P* = 0.5204). The median time from treatment to death for CNS methotrexate users was 11 months (range 1 – 28) compared with 9 months (range 1 – 24) for other methotrexate users (*P* = 0.9358). CNS IVL patients who used methotrexate (*n* = 6) had a median time from symptom to death of 15 months (range 9–28) versus 16 months (range 1–58) for CNS IVL patients who did not use methotrexate (*P* = 0.9469). Likewise, the median overall survival for CNS IVL patients on methotrexate was 14 months (range 1–28) versus 12 months (range 0–58) for CNS IVL patients not on methotrexate (*P* = 0.9246). Finally, the median time from treatment to death for CNS IVL patients who used methotrexate was 11 months (range 1–28) versus 10 months (range 0–58) for CNS IVL patients who did not use methotrexate (*P* = 0.9167).

### Geographical Outcomes of IVL

Central nervous system, PNS and other manifestations of ethnically Asian patients were 29.% and 71.% respectively. CNS, PNS and other manifestations of ethnically Non-Asian patients were 47 %, 2 %, and 51 % respectively. PNS cases were eliminated from comparison due to small sample size.

Asian (*n* = 34) and non-Asian (*n* = 43) CNS patients were compared. The geographic differences failed to become statistically significant (*p* > 0.05). IVL of other locations among Asian and Non- Asian patients were compared and determined statistically insignificant as well (*p* > 0.05).

### CNS complications

Cognitive impairment (including dementia and encephalopathy) was the most common presenting symptom and was reported in 168 (60.9 %) cases. Extremity paralysis (upper motor neuron) was seen in 61 (22.1 %) patients. Paralysis (upper motor neuron) included cases of single extremity paralysis, hemiplegia, and quadriplegia. Thirty-seven (13.4 %) patients presented with seizures including myoclonic jerks and status epilepticus. CNS complications are summarized in Table 1.

**Table 1:** Central nervous system (CNS) clinical presentations of intravascular lymphoma

CNS Symptoms	Number	Percent
Cognitive impairment/Dementia	168	60.90 %
Paralysis/paraplegia	61	22.1
Seizures	37	13.41 %
Vision disturbances	24	8.69 %
Ataxia	21	7.61 %
Stroke like symptoms	21	7.61 %
Headache	19	6.88 %
Myelopathy	16	5.78 %
Dysarthria	15	5.43 %
Sensory deficits	13	4.71 %
Hearing disturbances	12	4.35 %
Aphasia	11	3.99 %
Cranial Nerve Palsies	10	3.62 %
Dizziness/vertigo	8	2.99 %
Hyperreflexia	7	2.54 %
Motor deficits	5	1.81 %
Panhypopiutarism	5	1.81 %
Syncope	5	1.81 %
Psychosis	4	1.45 %
Antehypophyseal Failure	2	0.72 %
Positive Babinski	2	0.72 %
Dysphagia	2	0.72 %
Apraxia	2	0.72 %
Coma	1	0.36 %
Dystonia	1	0.36 %

### PNS complications

The most common symptoms involving the PNS were those of muscle weakness 37 (59.7 %), neurogenic bladder 23 (37.1 %), and neuropathies 17 (27.5 %). Cauda equina syndrome and conus medullaris syndrome were seen in 9 (14.5 %) patients. Saddle anesthesia commonly accompanying neurogenic bladder was seen in 6 (9.7 %) cases. Table 2 summarizes some of the PNS symptoms that IVL can mimic.

**Table 2:** Peripheral nervous system (PNS) clinical presentations of intravascular lymphoma

PNS Symptoms	Number	Percent
Muscle Weakness/Numbness	37	59.70 %
Neurogenic Bladder/Incontinence	23	37.10 %
Paresthesia’s	10	16.13 %
Cauda Equina Syndrome/Conus Medullaris Syndrome	9	14.52 %
Saddle Anesthesia	6	9.68 %
Neuropathies	5	8.10 %
Horner’s Syndrome	4	6.45 %
Polyradiculopathy/Radiculopathy	4	6.45 %
Guillen Barre	2	3.22 %
Myopathies	1	1.63 %

### Immunological assessment differences

It has been shown that tumors such as nasopharyngeal carcinomas are more commonly associated with Epstein-Barr virus (EBV) infections in Asian patients [9]. Out of 84 Asian patients with CNS manifestations, 28 patients did not know their EBV status. Twenty patients did not have either EBV1 or 2. Eight patients (14 %) were positive for EBV1, and 28 (50 %) were positive for EBV 2. Out of the 203 non-Asian CNS patients, 55 did not have their EBV status checked. Sixty three or 43 % of patients did not have EBV 1 or 2. Only 2 patients or 0.01 % were positive for EBV1 and 83 or 56 % were positive for EBV2. The results regarding EBV testing are summarized in Table 3. These findings might indicate an association between EBV2 and CNS IVL.

**Table 3:** Epstein-Barr Virus (EBV) immunological results of intravascular lymphoma

	Asian CNS	Asian PNS	Asian Other	Non-Asian CNS	Non-Asian PNS	Non-Asian Other
Total Patients	84	1	216	203	9	219
EBV Unknown	28	0	52	55	6	48
EBV Absent	20 (35 %)	0	58 (35 %)	63 (43 %)	2 (66 %)	77 (45 %)
EBV 1	8 (14 %)	0	19 (12 %)	2 (0.01 %)	0	9 (0.05 %)
EBV 2	28 (50.0 %)	1 (100 %)	87 (53 %)	83 (56 %)	1 (34 %)	85 (50 %)

*CNS* central nervous system

*PNS* peripheral nervous system

## Discussion

Our study population, spanning five decades, consisted of 654 patients. The majority of them (338 or 52 %) were presenting with neurological symptoms; 276 patients (42 %) presented with CNS symptoms and 62 (9.4 %) presented with PNS symptoms. According to geographical origin and variable clinical presentations, two main types of IVLs are identified; they are the Asian and non-Asian subtypes. As shown earlier, brain and skin were the most common organs affected in both of them [10]. However, occurrences of IVL between these two ethnicities failed to show statistical significance during our earlier analysis [6].

In this report, we compared the site of first organ affected between Asian and Non-Asian patients. During that analysis, we compared CNS and other sites of Asian and non-Asian patients. Time from initial symptoms to death of Asian and non-Asian patients was the same whether the initial presentation was at CNS or elsewhere (*p* > 0.05, Table 4). Overall survival of Asian or non-Asian patients was the same whether the initial presentation was at CNS or elsewhere (*p* > 0.05, Table 5). Treatment did not change their survival time (*p* > 0.05, Table 6). Murase and Nakamura reported that cutaneous and CNS manifestations were common, but not extensively observed in Asian IVL cases. They attributed their observation to the minimal or lack of neurological findings in the clinical presentation of these patients [11]. Our comparisons did not support a difference between Asian versus non-Asian patients.

**Table 4:** Comparison of asian and non-asian patients’ overall survival

Site	Asian/Non-Asian	Time from Symptom to Death (months)	*P*-value
CNS	Non-Asian	14 (1-54) *n* = 43	0.9305
	Asian	18 (1-58) *n* = 34	
Other	Non-Asian	15 (0-87) *n* = 100	0.7208
	Asian	14 (1-110) *n* = 93	
Site	Asian/Non-Asian	Overall Survival (months)	*P*-value
CNS	Non-Asian	10 (0-52) *n* = 43	0.4146
	Asian	15 (0-58) *n* = 34	
Other	Non-Asian	11 (0-84) *n* = 100	0.5446
	Asian	11 (0-96) *n* = 93	
Site	Asian/Non-Asian	Time from Treatment to Death (months)	*P*-value
CNS	Non-Asian	10 (0-52) *n* = 43	0.2858
	Asian	15 (0-58) *n* = 34	
Other	Non-Asian	10 (0-84) *n* = 100	0.2597
	Asian	11 (0-96) *n* = 93

**Table 5:** Comparisons of effect of methotrexate use

Methotrexate Use	Time from Symptom to Death (months)	*P*-value
Yes	15 (9 - 28) *n* = 6	0.9469
No + N/A	16 (1 - 58) *n* = 71	
Methotrexate Use	Overall Survival (months)	*P*-value
Yes	14 (1 - 28) *n* = 6	0.9246
No + N/A	12 (0 - 58) *n* = 71	
Methotrexate Use	Time from Treatment to Death (months)	*P*-value
Yes	11 (1 - 28) *n* = 6	0.9167
No + N/A	10 (0 - 58) *n* = 71

**Table 6:** Comparisons of effect of Methotrexate use

Methotrexate Use	Time from Symptom to Death (months)	*P*-value
Yes	17.0 (2.4-55.0) *n* = 18	0.5154
No	14.0 (0.4-110.0) *n* = 249	
Yes	17.0 (2.4-55.0) *n* = 18	0.4442
No + N/A	14.0 (0.4-110.0) *n* = 258	
No	14.0 (0.4-110.0) *n* = 249	0.5887
Yes + N/A	16.0 (1.7-55.0) *n* = 27	
Methotrexate Use	Overall Survival (months)	*P*-value
Yes	15.5 (1.0-44.0) *n* = 18	0.3677
No	11.0 (0.1-96.0) *n* = 249	
Yes	15.5 (1.0-44.0) *n* = 18	0.3164
No + N/A	10.1 (0.1-96.0) *n* = 258	
No	11.0 (0.1-96.0) *n* = 249	0.9292
Yes + N/A	10.0 (1.0-44.0) *n* = 27	
Methotrexate Use	Time from Treatment to Death (months)	*P*-value
Yes	13.0 (1.0-44.0) *n* = 18	0.7858
No	10.1 (0.1-96.0) *n* = 249	
Yes	13.0 (1.0-44.0) *n* = 18	0.7208
No + N/A	10.0 (0.1-96.0) *n* = 258	
No	10.1 (0.1-96.0) *n* = 249	0.5852
Yes + N/A	9.0 (1.0-44.0) *n* = 27	

The neurological symptoms varied according to the affected brain area [12]. Most of the pathology seemed to occur from ischemia due to vascular obstruction [13]. Among CNS symptoms of IVL, the most common presentation was cognitive impairment/dementia, which was seen in 60.9 % of patients. Stroke-like symptoms were explained more elaborately in earlier publications [2, 5, 14]. However, our analysis indicated that stroke-like symptoms were seen in only in 7.68 % or 21 patients. During most of those encounters, these patients were suspected of having strokes and valuable treatment time was lost. Presentations of these patients were similar to a stroke but clinical progression was rapid which can also be explained by multiple or multifocal strokes. Such a rapid presentation does not usually leave room for clinical suspicion of IVL, therefore diagnosis of CNS IVL can be missed.

Paralysis (22.1 %) and seizures (13.4 %), the second and third most common presentations, respectively, were also seen more frequently than stroke-like symptoms. Movement disorders following IVL were rare (0.36 %). Although localized and less functionally limiting relative to a stroke, neurological manifestations such as aphasia, apraxia, paralysis and paraplegia can also be included in the stroke category. Even after such amalgamation, the total number of patients who presented in that broad category was only 37 %, much lower than the patients who presented with cognitive impairments. We admit that IVL is rare; however, when a patient presents with rapidly progressive neurological manifestations or neurological manifestations that cannot be explained, IVL should be suspected [15]. Findings from Glass et al also favored cerebrovascular infarction (76 %) or stroke-like symptoms more than sub-acute encephalopathy’s (27 %). Mental status changes which can manifest with or without focal signs were categorized under sub-acute encephalopathies [15]. One reason for the difference in clinical manifestations was perhaps the smaller number of patients in the study by Glass et al [15].

The most common PNS symptoms are muscle weakness (59.7 %), neurogenic bladder (37.1 %), and neuropathies (27.5 %), respectively. Although 62 patients (9.4 %) had PNS symptoms, there were only 10 patients (0.01 %) with IVL confined to PNS locations. The evolution of neurological deficits is such that devastating CNS involvement is usually followed by PNS involvement. Tumor predilection for CNS vasculature relative to PNS can be a reason for this rapid migration. Seemingly, fewer patients with PNS symptoms support this hypothesis. Most of the PNS symptoms can be explained by CNS involvement rather than primary PNS involvement per se. Direct or indirect peripheral nerve involvement of IVL can mimic many peripheral nerve system disease presentations and therefore confuse clinicians (Table 3). The most frequent peripheral nervous system manifestation was muscle weakness. Tumor cell obstruction of moderate- to small-sized peripheral vessels supplying large mixed nerve roots can give rise to such a presentation. Similar IVL tumor cells were also identified at distal nerve targets in between nerve roots and muscles. However, the diagnosis of those was debated between IVL and neurolymphomatosis (NL) although both disease entities show a close relationship [16, 17]. Such observations warrant a more comprehensive study of IVL tumor cells at the molecular level. A few patients, who were initially diagnosed when presenting with PNS symptoms and treated in a timely fashion, survived before devastating CNS symptoms emerged. Therefore, clinical suspicion of IVL when patients present with PNS symptoms can potentially halt the unrelenting neurologic deterioration in a clinically meaningful way.

Earlier studies pointed out that various clinical presentations and lack of diagnostic algorithms are reasons why more than 50 % of patients with IVL are diagnosed postmortem [18]. We attempted to draw a disease progression pattern of nervous system IVL. We were unsuccessful due to the rapid progression of the disease as well as under reporting of the data. Therefore, until more elaborate clinical diagnostic criteria are drawn, care givers and clinicians should suspect IVL especially when patients show rapid progression of CNS symptoms, or unexplained CNS, PNS symptoms. More elaborate reporting of cases and diagnostic tests will also aid in the prompt diagnosis and treatment of this disease.

A study from Ferreri et al showed that patients with IVL limited to the skin had better survival. They attributed the early diagnosis of the cutaneous variant to be the main reason for this observation [10]. On the other hand, 60 % of the CNS IVL diagnoses are made postmortem [6]. Therefore prompt diagnosis and treatment is the key to turning the tables on CNS, PNS IVL. Out of the patients who were diagnosed ante mortem, 69 % survived 8.7 + 1.4 months and 29 % were alive at the time (range 5–48 months) their respective case reports were written. These calculations favor better survival if prompt diagnosis of CNS IVL is made. Absence of uniform radiological changes is a huge setback in achieving the goal of rapid diagnosis. Although likely to derive false negative results, MRI of the CNS should be routinely performed on patients with a suspicion of IVL to rule out more common types of neurologic diseases, and to complete staging work up for later management of the patient. Still, in the absence of neuro-radiological findings, a high index of clinical suspicion and a timely neurosurgical biopsy appear to be the common aspects of achieving a diagnosis of IVL. Since biopsy is an invasive test, it should be used sparingly.

We found no systematic studies confirming that CNS IVL has a preference for frontal brain regions. However, our observations show that the most frequent neurological finding following IVL is cognitive impairment. Human frontal and temporal lobes are relatively more responsible for cognition than other parts of the brain. Based on that observation, we hypothesize that CNS IVL might have a preference to those areas. Same organ IVL recurrences have been observed at a higher rate in CNS than in other organs [6]. Such a pattern also supports the intrinsic biological differences between CNS IVL versus the other organs. Few case reports exist of patients who have had ante mortem brain biopsies. Some were done on the frontal, cortical areas and they were positive for IVL [19]. Since IVL cannot be diagnosed solely based on radiography alone, identifying targets for biopsy will allow early diagnosis and management. Finally, the World Health Organization has classified this disorder as a sub-type of large B-cell lymphoma [3]. However, T-cell variants are rarely reported [20]. Most of the immunological markers used were B-cell markers and the comparison derived no new insights into the disease.

Fortunately, most of the cases of IVL that we reviewed were sensitive to systemic chemotherapy. We have previously reported that the addition of rituximab has more than doubled, and the combination of rituximab and doxorubicin has tripled the median survival rates of the same patient cohort [6]. It has also been shown that methotrexate is the most effective drug against primary central nervous system lymphomas [21, 22]. During our comparisons, methotrexate treatment did not change time from initial symptom to death, time from treatment to death or overall survival time (*p* > 0.05, Table 6). However, other studies geared more to test treatment effectiveness than ours showed that, methotrexate produces a response rate of 52–88 % as monotherapy and this response rate increases drastically to 70–94 % when combined with other medications [23–25]. High doses of methotrexate combined with rituximab and doxorubicin derive better results [6]. However, recent reports indicate methotrexate-resistant strains are emerging [26]. This is concerning because of our limited understanding of IVL. We should try to increase our knowledge base of IVL before resistance becomes rampant.

Most of the neurologic presentations have only been described in case studies. Meta-analysis of existing cases and case series was the only way to fully characterize the spectrum of neurologic presentations. Our study comprised of clinical and pathological descriptions of case reports and case series. To our knowledge, this is the largest study of the neurologic presentations of IVL. However, this study has several limitations. Due to the rarity of this disease, prospective analysis could not be done and published cases did not have uniform reporting criteria. There are also inherent biases in meta-analysis studies and great precaution was taken in order to eliminate any bias that could affect the results as explained in the methods section. At a minimum, we hope that our analysis will serve as a basis for future prospective studies of CNS IVL.

## Conclusion

IVL is a rare aggressive systemic B-cell lymphoma which over time can obstruct arterial blood flow to distal locations causing ischemia in various organs and subsequently systemic symptoms, neurological deficits or both. This study population consisted of 654 patients with IVL spanning over five decades. The aim of this study was to document the spectrum of clinical symptoms and their respective frequencies to help create a clinical framework for the prompt diagnosis of IVL. The majority of cases showed neurological symptoms (52 % of patients in this study). Of the patients presenting with neurological symptoms, the most common presentation was cognitive impairment/dementia (60.9 %). Paralysis (22.1 %) and seizures (13.4 %) were the second and third most common neurological presentations which were seen more frequently than stroke-like presentations (7.8 %). We did not find significant differences between Asian and non-Asian populations in regard to time from initial symptoms to death, overall survival or type of treatment. Due to the rarity of this tumor and lack of sensitive diagnostic methods, it is difficult to diagnose IVL in a timely fashion and therefore proper understanding of the disease presentation will allow early diagnosis and prompt intervention. Due to the findings in this study, we conclude that when a patient presents with rapidly progressive neurological manifestations or neurological manifestations that cannot be explained, IVL should be suspected. At a minimum, we hope that our analysis will serve as a basis for future prospective studies of CNS IVL.

## Additional files

Additional file 1:
**Table S1.** Bibliography for supplemental table 1A. (PDF 303 kb)
