# Supplementary material for: Neurological presentations of intravascular lymphoma (IVL): meta-analysis of 654 patients
Source: BMC Neurol. 2016 Jan 16;16:9. doi: 10.1186/s12883-015-0509-8 (PMC4744383; doi:10.1186/s12883-015-0509-8)
Supplement: Additional file 1: — Table S1. Bibliography for supplemental table 1A. (PDF 303 kb) [file 12883_2015_509_MOESM1_ESM.pdf]

Supplemental Table 1B. Bibliography for supplemental table 1A

[1-246]

1. Abraksia S, Kumar PD, Kasal J: **Two unusual lymphomas. Case 1: primary malignant lymphoma (diffuse large B-cell lymphoma) of the spleen mimicking splenic abscess**. *Journal of clinical oncology : official journal of the American Society of Clinical Oncology* 2000, **18**(21):3731-3733.
2. Adam DN, Beleznay KM, Randhawa RS, Marton M, Zhou Y: **Review of intravascular lymphoma with a report of treatment with allogenic peripheral blood stem cell transplant**. *Cutis; cutaneous medicine for the practitioner* 2008, **82**(4):267-272.
3. Agar JW, Gates PC, Vaughan SL, Machet D: **Renal biopsy in angiotropic large cell lymphoma**. *American journal of kidney diseases : the official journal of the National Kidney Foundation* 1994, **24**(1):92-96.
4. al-Chalabi A, Sivakumaran M, Holton J, West KP, Wood JK, Abbott RJ: **A case of intravascular malignant lymphomatosis (angiotropic lymphoma) with raised perinuclear antineutrophil cytoplasmic antibody titres--a hitherto unreported association**. *Clinical and laboratory haematology* 1994, **16**(4):363-369.
5. al-Hazzaa SA, Green WR, Mann RB: **Uveal involvement in systemic angiotropic large cell lymphoma. Microscopic and immunohistochemical studies**. *Ophthalmology* 1993, **100**(6):961-965.
6. Al-Humaidan H, Ali A, Ezzat A: **Intravascular large-cell lymphoma: Report of an unusual case**. *Annals of Saudi medicine* 1998, **18**(6):553-555.
7. al-Izzi MS, Sidhu PS, Garside PJ, Menai-Williams R: **Angiotropic large cell lymphoma (angioendotheliomatosis) presenting with protein-losing enteropathy**. *Postgraduate medical journal* 1988, **64**(750):313-314.
8. Albrecht R, Krebs B, Reusche E, Nagel M, Lencer R, Kretzschmar HA: **Signs of rapidly progressive dementia in a case of intravascular lymphomatosis**. *European archives of psychiatry and clinical neuroscience* 2005, **255**(4):232-235.
9. Amagasaki K, Yamazaki H, Ohmori K, Koizumi H, Hashizume K, Sasaguchi N: **Malignant intravascular lymphomatosis associated with venous stenosis. Case report**. *Journal of neurosurgery* 1999, **90**(2):355-358.
10. Anda T, Haraguchi W, Miyazato H, Tanaka S, Ishihara T, Aozasa K, Nakamichi I: **Ruptured distal middle cerebral artery aneurysm filled with tumor cells in a patient with intravascular large B-cell lymphoma**. *Journal of neurosurgery* 2008, **109**(3):492-496.
11. Anghel G, Petrinato G, Severino A, Remotti D, Insabato L, De Renzo A, Rotoli B, Majolino I: **Intravascular B-cell lymphoma: report of two cases with different clinical presentation but rapid central nervous system involvement**. *Leukemia & lymphoma* 2003, **44**(8):1353-1359.
12. Angioi K, Bodaghi B, Kaminsky P, Mokhtari K, Lubetzki C, LeHoang P: **Intravascular lymphoma mimicking a Vogt-Koyanagi-Harada disease**. *Ocular immunology and inflammation* 2011, **19**(2):132-134.
13. Ansbacher L, Low N, Beck D, Boarini D, Jacoby C, Cancilla PA: **Neoplastic angioendotheliosis: a clinicopathological entity with multifocal presentation. Case report**. *Journal of neurosurgery* 1981, **54**(3):412-415.

14. Ansell J, Bhawan J, Cohen S, Sullivan J, Sherman D: **Histiocytic lymphoma and malignant angioendotheliomatosis: one disease or two?** *Cancer* 1982, **50**(8):1506-1512.
15. Aoki A, Okamura M, Ueda A, Ohno S, Hagiwara E, Tsuji T, Misumi M, Kawachi K, Sasaki T, Inoue Y *et al*: **An autopsy case of intravascular lymphomatosis with dermatomyositis**. *Internal medicine* 2002, **41**(3):241-244.
16. Aoki Y, Takamiya M, Satoh T, Fujita S, Kato H, Maeno Y: **A fatal case of hemoperitoneum after ultrasound-guided liver biopsy in a patient with intravascular large B-cell lymphoma**. *Legal medicine* 2011, **13**(4):191-195.
17. Aouba A, Diop S, Saadoun D, Trebbia G, Vilde F, Patri B, Hermine O: **Severe pulmonary arterial hypertension as initial manifestation of intravascular lymphoma: case report**. *American journal of hematology* 2005, **79**(1):46-49.
18. Arboix A, Costa I, Besses C, Sans-Sabrafen J: **Acute pseudobulbar palsy as the initial presentation of intravascular lymphomatosis**. *European journal of internal medicine* 2004, **15**(2):128-130.
19. Arnn ET, Yam LT, Li CY: **Systemic angioendotheliomatosis presenting with hemolytic anemia**. *American journal of clinical pathology* 1983, **80**(2):246-251.
20. Asagoe K, Fujimoto W, Yoshino T, Mannami T, Liu Y, Kanzaki H, Arata J: **Intravascular lymphomatosis of the skin as a manifestation of recurrent B-cell lymphoma**. *Journal of the American Academy of Dermatology* 2003, **48**(2 Suppl):S1-4.
21. Askarian F, Xu D: **Adrenal enlargement and insufficiency: a common presentation of intravascular large B-cell lymphoma**. *American journal of hematology* 2006, **81**(6):411-413.
22. Au WY, Shek WH, Nicholls J, Tse KM, Todd D, Kwong YL: **T-cell intravascular lymphomatosis (angiotropic large cell lymphoma): association with Epstein-Barr viral infection**. *Histopathology* 1997, **31**(6):563-567.
23. Axelsen RA, Laird PP, Horn M: **Intravascular large cell lymphoma: diagnosis on renal biopsy**. *Pathology* 1991, **23**(3):241-243.
24. Aznar AO, Montero MA, Rovira R, Vidal FR: **Intravascular large B-cell lymphoma presenting with neurological syndromes: clinicopathologic study**. *Clinical neuropathology* 2007, **26**(4):180-186.
25. Baehring JM, Longtine J, Hochberg FH: **A new approach to the diagnosis and treatment of intravascular lymphoma**. *Journal of neuro-oncology* 2003, **61**(3):237-248.
26. Bai X, Li X, Wan L, Wang G, Jia N, Geng J: **Intravascular large B-cell lymphoma of the kidney: a case report**. *Diagnostic pathology* 2011, **6**:86.
27. Balkema C, Meersseman W, Hermans G, Stroobants S, Verhoef G, Verbeken E, Demaerel P, Blockmans D: **Usefulness of FDG-PET to diagnose intravascular lymphoma with encephalopathy and renal involvement**. *Acta clinica Belgica* 2008, **63**(3):185-189.
28. Banerjee SS, Harris M: **Angiotropic lymphoma presenting in the prostate**. *Histopathology* 1988, **12**(6):667-670.
29. Baraniskin A, Jahnert A, Ahle G, Theile A, Kretzschmar H, Schmiegel W, Schroers R: **Intravascular large B-cell lymphoma presenting as dementia and hemolytic anemia**. *Onkologie* 2010, **33**(4):174-177.

30. Barnett CR, Seo S, Husain S, Grossman ME: **Intravascular B-cell lymphoma: the role of skin biopsy**. *The American Journal of dermatopathology* 2008, **30**(3):295-299.
31. Bauer A, Perras B, Sufke S, Horny HP, Kreft B: **Myocardial infarction as an uncommon clinical manifestation of intravascular large cell lymphoma**. *Acta cardiologica* 2005, **60**(5):551-555.
32. Baum CL, Stone MS, Liu V: **Atypical intravascular CD30+ T-cell proliferation following trauma in a healthy 17-year-old male: first reported case of a potential diagnostic pitfall and literature review**. *Journal of cutaneous pathology* 2009, **36**(3):350-354.
33. Baumann TP, Hurwitz N, Karamitopolou-Diamantis E, Probst A, Herrmann R, Steck AJ: **Diagnosis and treatment of intravascular lymphomatosis**. *Archives of neurology* 2000, **57**(3):374-377.
34. Bazhenova L, Higginbottom P, Mason J: **Intravascular lymphoma: a role for single-agent rituximab**. *Leukemia & lymphoma* 2006, **47**(2):337-341.
35. Ben-Ezra J, Sheibani K, Kendrick FE, Winberg CD, Rappaport H: **Angiotropic large cell lymphoma of the prostate gland: an immunohistochemical study**. *Human pathology* 1986, **17**(9):964-967.
36. Berger JR, Jones R, Wilson D: **Intravascular lymphomatosis presenting with sudden hearing loss**. *Journal of the neurological sciences* 2005, **232**(1-2):105-109.
37. Berger TG, Dawson NA: **Angioendotheliomatosis**. *Journal of the American Academy of Dermatology* 1988, **18**(2 Pt 2):407-412.
38. Bergmann M, Terzija-Wessel U, Blasius S, Kuchelmeister K, Kryne-Kubat B, Gerhard L, Beneicke U, Berlit P: **Intravascular lymphomatosis of the CNS: clinicopathologic study and search for expression of oncoproteins and Epstein-Barr virus**. *Clinical neurology and neurosurgery* 1994, **96**(3):236-243.
39. Beristain X, Azzarelli B: **The neurological masquerade of intravascular lymphomatosis**. *Archives of neurology* 2002, **59**(3):439-443.
40. Bhagwati NS, Oiseth SJ, Abebe LS, Wiernik PH: **Intravascular lymphoma associated with hemophagocytic syndrome: a rare but aggressive clinical entity**. *Annals of hematology* 2004, **83**(4):247-250.
41. Bhawan J, Wolff SM, Ucci AA, Bhan AK: **Malignant lymphoma and malignant angioendotheliomatosis: one disease**. *Cancer* 1985, **55**(3):570-576.
42. Bogomolski-Yahalom V, Lossos IS, Okun E, Sherman Y, Lossos A, Polliack A: **Intravascular lymphomatosis--an indolent or aggressive entity?** *Leukemia & lymphoma* 1998, **29**(5-6):585-593.
43. Boslooper K, Dijkhuizen D, van der Velden AW, Dal M, Meilof JF, Hoogenberg K: **Intravascular lymphoma as an unusual cause of multifocal cerebral infarctions discovered on FDG-PET/CT**. *The Netherlands journal of medicine* 2010, **68**(6):261-264.
44. Bots GT: **Angioendotheliomatosis of the central nervous system**. *Acta neuropathologica* 1974, **28**(1):75-78.
45. Bouzani M, Karmiris T, Rontogianni D, Delimpassi S, Apostolidis J, Mpakiri M, Nikiforakis E: **Disseminated intravascular B-cell lymphoma: clinicopathological features and outcome of three cases treated with**

anthracycline-based immunochemotherapy. *The oncologist* 2006, **11**(8):923-928.
46. Bozzoli V, Tisi MC, D'Alo F, Massini G, Mansueto G, Vannata B, Arena V, Larocca LM, Teofili L, Leone G *et al*: **Intravascular large B cell lymphoma: when lymphoma is suspected but routine diagnostic work-up is negative**. *Leukemia & lymphoma* 2009, **50**(11):1900-1903.
47. Byard RW, Orizaga M: **Neoplastic angioendotheliomatosis involving the central nervous system**. *The Canadian journal of neurological sciences Le journal canadien des sciences neurologiques* 1986, **13**(4):348.
48. Calamia KT, Miller A, Shuster EA, Perniciaro C, Menke DM: **Intravascular lymphomatosis. A report of ten patients with central nervous system involvement and a review of the disease process**. *Advances in experimental medicine and biology* 1999, **455**:249-265.
49. Carroll TJ, Jr., Schelper RL, Goeken JA, Kemp JD: **Neoplastic angioendotheliomatosis: immunopathologic and morphologic evidence for intravascular malignant lymphomatosis**. *American journal of clinical pathology* 1986, **85**(2):169-175.
50. Carter DK, Batts KP, de Groen PC, Kurtin PJ: **Angiotropic large cell lymphoma (intravascular lymphomatosis) occurring after follicular small cleaved cell lymphoma**. *Mayo Clinic proceedings Mayo Clinic* 1996, **71**(9):869-873.
51. Cerroni L, Massone C, Kutzner H, Mentzel T, Umbert P, Kerl H: **Intravascular large T-cell or NK-cell lymphoma: a rare variant of intravascular large cell lymphoma with frequent cytotoxic phenotype and association with Epstein-Barr virus infection**. *The American journal of surgical pathology* 2008, **32**(6):891-898.
52. Cerroni L, Zalaudek I, Kerl H: **Intravascular large B-cell lymphoma colonizing cutaneous hemangiomas**. *Dermatology* 2004, **209**(2):132-134.
53. Chakravarty K, Goyal M, Scott DG, McCann BG: **Malignant 'angioendotheliomatosis'--(intravascular lymphomatosis) an unusual cutaneous lymphoma in rheumatoid arthritis**. *British journal of rheumatology* 1993, **32**(10):932-934.
54. Chang A, Zic JA, Boyd AS: **Intravascular large cell lymphoma: a patient with asymptomatic purpuric patches and a chronic clinical course**. *Journal of the American Academy of Dermatology* 1998, **39**(2 Pt 2):318-321.
55. Chapin JE, Davis LE, Kornfeld M, Mandler RN: **Neurologic manifestations of intravascular lymphomatosis**. *Acta neurologica Scandinavica* 1995, **91**(6):494-499.
56. Chapman JE, Jr., Loy WA: **Cerebral angioendotheliomatosis associated with hemispheric symptoms of carotid arterial origin**. *Journal of vascular surgery* 1985, **2**(2):281-284.
57. Chaukiyal P, Singh S, Woodlock T, Dolan JG, Bruner K: **Intravascular large B-cell lymphoma with multisystem involvement**. *Leukemia & lymphoma* 2006, **47**(8):1688-1690.
58. Chen M, Qiu B, Kong J, Chen J: **Angiotropic T cell lymphoma**. *Chinese medical journal* 1998, **111**(8):762-764.

59. Chen P, Gaetjens E, Sher J, Somasundaram M, Lee TK, How SW, Cho NH: **Malignant angioendotheliomatosis manifesting as ascending spinal cord dysfunction**. *New York state journal of medicine* 1987, **87**(8):470-472.
60. Chen TM, Crow MK, Teller C: **Angiotropic large-cell lymphoma with striking blue plaques**. *Journal of the American Academy of Dermatology* 2003, **48**(4):633-634.
61. Chim CS, Choy C, Ooi GC, Chung LP, Wong KK, Liang R: **Two unusual lymphomas. Case 2: pulmonary intravascular lymphomatosis**. *Journal of clinical oncology : official journal of the American Society of Clinical Oncology* 2000, **18**(21):3733-3735.
62. Chim CS, Loong F: **Intravascular lymphomatosis of the prostate gland**. *British journal of haematology* 2002, **119**(1):2.
63. Chinen Y, Nakao M, Sugitani-Yamamoto M, Kiyota M, Horiike S, Kuroda J, Taniwaki M: **Intravascular B-cell lymphoma with hypercalcemia as the initial presentation**. *International journal of hematology* 2011, **94**(6):567-570.
64. Cho K, Kim C, Yang S, Kim B, Kim J: **Angiocentric T cell lymphoma of the skin presenting as inflammatory nodules of the leg**. *Clinical and experimental dermatology* 1997, **22**(2):104-108.
65. Chu P, Costa J, Lachman MF: **Angiotropic large cell lymphoma presenting as primary adrenal insufficiency**. *Human pathology* 1996, **27**(2):209-211.
66. Clark WC, Dohan FC, Jr., Moss T, Schweitzer JB: **Immunocytochemical evidence of lymphocytic derivation of neoplastic cells in malignant angioendotheliomatosis**. *Journal of neurosurgery* 1991, **74**(5):757-762.
67. Collins KA, Davis GJ: **Angiotropic large cell lymphoma**. *Southern medical journal* 1995, **88**(2):235-238.
68. Conlin PA, Orden MB, Hough TR, Morgan DL: **Myeloperoxidase-positive intravascular large B-cell lymphoma**. *Archives of pathology & laboratory medicine* 2001, **125**(7):948-950.
69. Csomor J, Kaszas I, Kollar B, Pajor L, Egyhazi Z, Fekete S, Egyed M, Timar B: **Prolonged survival using anti-CD20 combined chemotherapy in primary prostatic intravascular large B-cell lymphoma**. *Pathology oncology research : POR* 2008, **14**(3):281-284.
70. Curtis JL, Warnock ML, Conrad DJ, Helfend LK, Boushey HA: **Intravascular (angiotropic) large-cell lymphoma ('malignant angioendotheliomatosis') with small vessel pulmonary vascular obstruction and hypercalcemia**. *The Western journal of medicine* 1991, **155**(1):72-76.
71. D'Agati V, Sablay LB, Knowles DM, Walter L: **Angiotropic large cell lymphoma (intravascular malignant lymphomatosis) of the kidney: presentation as minimal change disease**. *Human pathology* 1989, **20**(3):263-268.
72. Daniel SE, Rudge P, Scaravilli F: **Malignant angioendotheliosis involving the nervous system: support for a lymphoid origin of the neoplastic cells**. *Journal of neurology, neurosurgery, and psychiatry* 1987, **50**(9):1173-1177.
73. Davis TS: **Intravascular lymphoma presenting with cauda equina syndrome: treated with CHOP and rituxan**. *Leukemia & lymphoma* 2003, **44**(5):887-888.

74. Debiais S, Bonnaud I, Cottier JP, Destrieux C, Saudeau D, de Toffol B, Arbion F, Benboubker L, Autret A: **A spinal cord intravascular lymphomatosis with exceptionally good outcome**. *Neurology* 2004, **63**(7):1329-1330.
75. Dedic K, Belada D, Zak P, Nozicka Z: **Intravascular large B-cell lymphoma presenting as cutaneous panniculitis**. *Acta medica (Hradec Kralove) / Universitas Carolina, Facultas Medica Hradec Kralove* 2003, **46**(3):121-123.
76. Deisch J, Fuda FB, Chen W, Karandikar N, Arbini AA, Zhou XJ, Wang HY: **Segmental tandem triplication of the MLL gene in an intravascular large B-cell lymphoma with multisystem involvement: a comprehensive morphologic, immunophenotypic, cytogenetic, and molecular cytogenetic antemortem study**. *Archives of pathology & laboratory medicine* 2009, **133**(9):1477-1482.
77. Demirer T, Dail DH, Aboulafia DM: **Four varied cases of intravascular lymphomatosis and a literature review**. *Cancer* 1994, **73**(6):1738-1745.
78. Deschamps L, Signate A, Delaunay C, Morales I, Warter A, Smadja D, Derancourt C: **A blind skin biopsy diagnosing an intravascular large B-cell lymphoma**. *European journal of dermatology : EJD* 2011, **21**(1):114-115.
79. Deusch E, Mayr A, Hobisch-Hagen P, Fend F, Mutz N, Bangerl I, Hasibeder W: **Angiotropic large B-cell lymphoma misdiagnosed as urosepsis with multiple organ dysfunction syndrome**. *Acta anaesthesiologica Scandinavica* 1999, **43**(1):100-103.
80. Devlin T, Moll S, Hulette C, Morgenlander JC: **Intravascular malignant lymphomatosis with neurologic presentation: factors facilitating antemortem diagnosis**. *Southern medical journal* 1998, **91**(7):672-676.
81. DiGiuseppe JA, Nelson WG, Seifter EJ, Boitnott JK, Mann RB: **Intravascular lymphomatosis: a clinicopathologic study of 10 cases and assessment of response to chemotherapy**. *Journal of clinical oncology : official journal of the American Society of Clinical Oncology* 1994, **12**(12):2573-2579.
82. Dominguez FE, Rosen LB, Kramer HC: **Malignant angioendotheliomatosis proliferans. Report of an autopsied case studied with immunoperoxidase**. *The American Journal of dermatopathology* 1986, **8**(5):419-425.
83. Domizio P, Hall PA, Cotter F, Amiel S, Tucker J, Besser GM, Levison DA: **Angiotropic large cell lymphoma (ALCL): morphological, immunohistochemical and genotypic studies with analysis of previous reports**. *Hematological oncology* 1989, **7**(3):195-206.
84. Dozic S, Suvakovic V, Cvetkovic D, Jevtovic D, Skender M: **Neoplastic angioendotheliomatosis (NAE) of the CNS in a patient with AIDS subacute encephalitis, diffuse leukoencephalopathy and meningo-cerebral cryptococcosis**. *Clinical neuropathology* 1990, **9**(6):284-289.
85. Drlicek M, Grisold W, Liszka U, Hitzenberger P, Machacek E: **Angiotropic lymphoma (malignant angioendotheliomatosis) presenting with rapidly progressive dementia**. *Acta neuropathologica* 1991, **82**(6):533-535.
86. Drobacheff C, Blanc D, Zultak M, Humbert P, Carbillet JP, Dupond JL, Laurent R: **Malignant angioendotheliomatosis. Reclassification as an angiotropic lymphoma**. *International journal of dermatology* 1989, **28**(7):454-456.

87. Duan X, Lapus A, Brown RE, Chen L: **Intravascular large B-cell lymphoma presenting as cholecystitis and pancytopenia: case report with literature review**. *Annals of clinical and laboratory science* 2011, **41**(3):262-266.
88. Dubas F, Saint-Andre JP, Pouplard-Barthelaix A, Delestre F, Emile J: **Intravascular malignant lymphomatosis (so-called malignant angioendotheliomatosis): a case confined to the lumbosacral spinal cord and nerve roots**. *Clinical neuropathology* 1990, **9**(3):115-120.
89. Dufau JP, Le Tourneau A, Molina T, Le Houcq M, Claessens YE, Rio B, Delmer A, Diebold J: **Intravascular large B-cell lymphoma with bone marrow involvement at presentation and haemophagocytic syndrome: two Western cases in favour of a specific variant**. *Histopathology* 2000, **37**(6):509-512.
90. Dunphy CH: **Primary cutaneous angiotropic large-cell lymphoma in a patient with acquired immunodeficiency syndrome**. *Archives of pathology & laboratory medicine* 1995, **119**(8):757-759.
91. Elner VM, Hidayat AA, Charles NC, Davitz MA, Smith ME, Burgess D, Dawson N: **Neoplastic angioendotheliomatosis. A variant of malignant lymphoma immunohistochemical and ultrastructural observations of three cases**. *Ophthalmology* 1986, **93**(9):1237-1245.
92. Emura I, Naito M, Wakabayashi M, Yoshizawa H, Arakawa M, Chou T: **Detection of circulating tumor cells in a patient with intravascular lymphomatosis: a case study examined by the cytology method**. *Pathology international* 1998, **48**(1):63-66.
93. Estalilla OC, Koo CH, Brynes RK, Medeiros LJ: **Intravascular large B-cell lymphoma. A report of five cases initially diagnosed by bone marrow biopsy**. *American journal of clinical pathology* 1999, **112**(2):248-255.
94. Evert M, Lehringer-Polzin M, Mobius W, Pfeifer U: **Angiotropic large-cell lymphoma presenting as pulmonary small vessel occlusive disease**. *Human pathology* 2000, **31**(7):879-882.
95. Feldmann R, Schierl M, Sittenthaler M, Jahn R, Wogritsch C, Cerroni L, Steiner A, Breier F: **Intravascular large B-cell lymphoma of the skin: typical clinical manifestations and a favourable response to rituximab-containing therapy**. *Dermatology* 2009, **219**(4):344-346.
96. Felizardo M, Mendes AC, Fernandes A, Campos P, Magalhaes V, Correia I, Pignatelli A, Ferreira C, Sotto-Mayor R, Bugalho de Almeida A: **Intravascular pulmonary lymphoma with good response to treatment. A case report**. *Revista portuguesa de pneumologia* 2008, **14**(6):857-868.
97. Ferry JA, Harris NL, Picker LJ, Weinberg DS, Rosales RK, Tapia J, Richardson EP, Jr.: **Intravascular lymphomatosis (malignant angioendotheliomatosis). A B-cell neoplasm expressing surface homing receptors**. *Modern pathology : an official journal of the United States and Canadian Academy of Pathology, Inc* 1988, **1**(6):444-452.
98. Ferry JA, Sohani AR, Longtine JA, Schwartz RA, Harris NL: **HHV8-positive, EBV-positive Hodgkin lymphoma-like large B-cell lymphoma and HHV8-positive intravascular large B-cell lymphoma**. *Modern pathology : an official journal of the United States and Canadian Academy of Pathology, Inc* 2009, **22**(5):618-626.

99. Fiegl M, Greil R, Pechlaner C, Krugmann J, Dirnhofer S: **Intravascular large B-cell lymphoma with a fulminant clinical course: a case report with definite diagnosis post mortem**. *Annals of oncology : official journal of the European Society for Medical Oncology / ESMO* 2002, **13**(9):1503-1506.

100. Fievez M, Fievez C, Hustin J: **Proliferating systematized angioendotheliomatosis**. *Archives of dermatology* 1971, **104**(3):320-324.

101. Flores-Vazquez F, de Leon-Bojorge B, Ortiz-Hidalgo C, Capurso M: **Intravascular lymphoma presenting with clinical features of cholecystitis**. *Southern medical journal* 2001, **94**(9):946-947.

102. Fozza C, Bonfigli S, Conti M, Dore F, Longinotti M: **Long-lasting fever of unknown origin preceding the diagnosis of intravascular lymphomatosis: a further case stimulates some remarks**. *American journal of hematology* 2003, **74**(3):211-213.

103. Fujiwara A, Nagayama S, Amada S, Shimamoto T, Shimao Y, Hayashi T: **Intravascular large B-cell lymphoma involving mainly the uterus: report of a case using liquid-based cytology of the endometrium**. *Acta cytologica* 2010, **54**(5 Suppl):787-792.

104. Fukuchi M, Fushimi S, Yoneya M, Hirayama A: **An autopsy case of intravascular malignant lymphoma presenting with intracranial B-cell type malignant lymphoma**. *Noshuyo byori = Brain tumor pathology* 1996, **13**(2):119-125.

105. Fukushima A, Okada Y, Tanikawa T, Onaka T, Tanaka A, Higashi T, Tsukada J, Tanaka Y: **Primary bilateral adrenal intravascular large B-cell lymphoma associated with adrenal failure**. *Internal medicine* 2003, **42**(7):609-614.

106. Fulling KH, Gersell DJ: **Neoplastic angioendotheliomatosis. Histologic, immunohistochemical, and ultrastructural findings in two cases**. *Cancer* 1983, **51**(6):1107-1118.

107. Gabor EP, Sherwood T, Mercola KE: **Intravascular lymphomatosis presenting as adult respiratory distress syndrome**. *American journal of hematology* 1997, **56**(3):155-160.

108. Ganeshan A, Soonawalla Z, De M, Baxter J: **Intravascular lymphoma: a diagnostic enigma**. *Journal of the Royal Society of Medicine* 2002, **95**(1):37-38.

109. Garg A, Hosfield EM, Brickner L: **Disseminated intravascular large B cell lymphoma with slowly decreasing high-density lipoprotein cholesterol**. *Southern medical journal* 2011, **104**(1):53-56.

110. Gaul C, Hanisch F, Neureiter D, Behrmann C, Neundorfer B, Winterholler M: **Intravascular lymphomatosis mimicking disseminated encephalomyelitis and encephalomyelopathy**. *Clinical neurology and neurosurgery* 2006, **108**(5):486-489.

111. Georgin-Lavialle S, Darmon M, Galicier L, Fysekidis M, Azoulay E: **Intravascular lymphoma presenting as a specific pulmonary embolism and acute respiratory failure: a case report**. *Journal of medical case reports* 2009, **3**:7253.

112. Ghorbani RP, Shokouh-Amiri H, Gaber LW: **Intragraft angiotropic large-cell lymphoma of T cell-type in a long-term renal allograft recipient**. *Modern*

pathology : an official journal of the United States and Canadian Academy of Pathology, Inc 1996, **9**(6):671-676.

113. Gill S, Melosky B, Haley L, ChanYan C: **Use of random skin biopsy to diagnose intravascular lymphoma presenting as fever of unknown origin**. *The American journal of medicine* 2003, **114**(1):56-58.
114. Gioulis M, Ben G, Iuzzolino P, De Biasi F, Marchini C, Zambito Marsala S: **Subacute cognitive disorders as initial presentation of intravascular lymphoma: a case report and review of literature**. *Neurological sciences : official journal of the Italian Neurological Society and of the Italian Society of Clinical Neurophysiology* 2010, **31**(3):369-372.
115. Glass J, Hochberg FH, Miller DC: **Intravascular lymphomatosis. A systemic disease with neurologic manifestations**. *Cancer* 1993, **71**(10):3156-3164.
116. Gleason BC, Brinster NK, Granter SR, Pinkus GS, Lindeman NI, Miller DM: **Intravascular cytotoxic T-cell lymphoma: A case report and review of the literature**. *Journal of the American Academy of Dermatology* 2008, **58**(2):290-294.
117. Go A, Venugopal P, Loew J, Djordjevic D: **Durable remission of intravascular lymphoma with central nervous system involvement following chemotherapy and rituximab**. *Clinical advances in hematology & oncology : H&O* 2006, **4**(6):439-441.
118. Goh SG, Chuah KL, Tan PH: **Intravascular lymphomatosis of the lung and liver following eyelid lymphoma in a Chinese man and review of primary pulmonary intravascular lymphomatosis**. *Pathology* 2002, **34**(1):82-85.
119. Grove CS, Robbins PD, Kermode AG: **Intravascular lymphoma presenting as progressive paraparesis**. *Journal of clinical neuroscience : official journal of the Neurosurgical Society of Australasia* 2008, **15**(9):1056-1058.
120. Gupta AK, Lipa M, Haberman HF: **Proliferating angioendotheliomatosis. Case with long survival and review of literature**. *Archives of dermatology* 1986, **122**(3):314-319.
121. Haber H, Harris-Jones JN, Wells AL: **Intravascular Endothelioma (Endothelioma in Situ, Systemic Endotheliomatosis)**. *Journal of clinical pathology* 1964, **17**:608-611.
122. Hadjileontis CG, Kostopoulos IS, Kaloutsi VD, Nikolaou AC, Kotoula VA, Papadimitriou CS: **An extremely rare case of synchronous occurrence in the larynx of intravascular lymphoma and in situ squamous cell carcinoma**. *Leukemia & lymphoma* 2003, **44**(6):1053-1057.
123. Hamada K, Hamada T, Satoh M, Tashiro K, Katoh I, Naganuma M, Shima K, Ogata A, Nagashima K: **Two cases of neoplastic angioendotheliomatosis presenting with myelopathy**. *Neurology* 1991, **41**(7):1139-1140.
124. Han K, Haley JC, Carlson K, Pinter-Brown L, Soriano T: **Regression of cutaneous intravascular lymphoma with rituximab**. *Cutis; cutaneous medicine for the practitioner* 2003, **72**(2):137-140.
125. Hanihara T, Takahashi T, Shimada T, Mizuguchi M, Yagishita S: **Parathyroid hormone-related protein-associated hypercalcemia in probable intravascular lymphoma of B-cell type**. *American journal of hematology* 1996, **53**(2):144-145.

126. Harris CP, Sigman JD, Jaeckle KA: **Intravascular malignant lymphomatosis: amelioration of neurological symptoms with plasmapheresis**. *Annals of neurology* 1994, **35**(3):357-359.
127. Hayashi T, Watanabe E, Ogawa M, Terakura M, Nonaka Y, Hirano M, Inoue K, Iizuka T, Oka H, Suzuki T *et al*: **Angiotropic B-cell lymphoma presenting with progressive dementia: an autopsy case and review of the literature in Japan**. *Internal medicine* 1995, **34**(11):1134-1139.
128. Heafield MT, Carey M, Williams AC, Cullen M: **Neoplastic angioendotheliomatosis: a treatable "vascular dementia" occurring in an immunosuppressed transplant patient**. *Clinical neuropathology* 1993, **12**(2):102-106.
129. Heinrich A, Vogelgesang S, Kirsch M, Khaw AV: **Intravascular lymphomatosis presenting as rapidly progressive dementia**. *European neurology* 2005, **54**(1):55-58.
130. Helm TN, Bergfeld WF, Elston D: **Angiotropic lymphoma: malignant angioendotheliomatosis**. *Cutis; cutaneous medicine for the practitioner* 1992, **50**(3):204-206.
131. Hishikawa N, Niwa H, Hara T, Hara K, Ito M, Shimada S, Yoshida M, Hashizume Y, Murakami N: **An autopsy case of lymphomatosis cerebri showing pathological changes of intravascular large B-cell lymphoma in visceral organs**. *Neuropathology : official journal of the Japanese Society of Neuropathology* 2011, **31**(6):612-619.
132. Hofman MS, Fields P, Yung L, Mikhaeel NG, Ireland R, Nunan T: **Meningeal recurrence of intravascular large B-cell lymphoma: early diagnosis with PET-CT**. *British journal of haematology* 2007, **137**(5):386.
133. Holmes NE, Gordon CL, Lightfoot N, Crowley P, Buchanan RR, Smith CL, Johnson PD: **Intravascular large B cell lymphoma: an elusive cause of pyrexia of unknown origin diagnosed postmortem**. *Clinical infectious diseases : an official publication of the Infectious Diseases Society of America* 2010, **51**(9):e61-64.
134. Holmoy T, Nakstad PH, Fredo HL, Kumar T: **Intravascular large B-cell lymphoma presenting as cerebellar and cerebral infarction**. *Archives of neurology* 2007, **64**(5):754-755.
135. Horvath B, Demeter J, Eros N, Harsing J, Csomor J, Matolcsy A, Bottlik G, Gyori G, Marschalko M, Karpati S: **Intravascular large B-cell lymphoma: remission after rituximab-cyclophosphamide, doxorubicin, vincristine, and prednisolone chemotherapy**. *Journal of the American Academy of Dermatology* 2009, **61**(5):885-888.
136. Hoshino A, Kawada E, Ukita T, Itoh K, Sakamoto H, Fujita K, Mantani N, Kogure T, Tamura J: **Usefulness of FDG-PET to diagnose intravascular lymphomatosis presenting as fever of unknown origin**. *American journal of hematology* 2004, **76**(3):236-239.
137. Hsiao CH, Su IJ, Hsieh SW, Huang SF, Tsai TF, Chen MY, How SW: **Epstein-Barr virus-associated intravascular lymphomatosis within Kaposi's sarcoma in an AIDS patient**. *The American journal of surgical pathology* 1999, **23**(4):482-487.

138. Hsieh MS, Yeh YC, Chou YH, Lin CW: **Intravascular large B cell lymphoma in Taiwan: an Asian variant of non-germinal-center origin**. *Journal of the Formosan Medical Association = Taiwan yi zhi* 2010, **109**(3):185-191.
139. Hsu YH, Tseng BY, Shyu WC, Yen PS: **Intravascular lymphomatosis mimicking acute disseminated encephalomyelitis: a case report**. *The Kaohsiung journal of medical sciences* 2005, **21**(2):93-97.
140. Hundsberger T, Cogliatti S, Kleger GR, Fretz C, Gahler A, Anliker M, Fournier JY, von Moos R, Tettenborn B, Driessen C: **Intravascular lymphoma mimicking cerebral stroke: report of two cases**. *Case reports in neurology* 2011, **3**(3):278-283.
141. Hwang WS, Jung CW, Ko YH, Seo SW, Na DL: **Intravascular lymphomatosis presenting as acute hemispheric dysfunction**. *Journal of stroke and cerebrovascular diseases : the official journal of National Stroke Association* 2012, **21**(8):904 e901-902.
142. Iijima M, Fujita A, Uchigata M, Katoo H: **Change of brain MRI findings in a patient with intravascular malignant lymphomatosis**. *European journal of neurology : the official journal of the European Federation of Neurological Societies* 2007, **14**(5):e4-5.
143. Im SH, Lee SY, Kim NH: **Headache as the only presentation of intravascular lymphoma**. *Headache* 2008, **48**(4):627-629.
144. Imai H, Kajimoto K, Taniwaki M, Miura I, Hatta Y, Hashizume Y, Watanabe M, Shiraishi T, Nakamura S: **Intravascular large B-cell lymphoma presenting with mass lesions in the central nervous system: a report of five cases**. *Pathology international* 2004, **54**(4):231-236.
145. Imamura K, Awaki E, Aoyama Y, Kondo S, Horie Y, Ohama E, Nakashima K: **Intravascular large B-cell lymphoma following a relapsing stroke with temporary fever: a brain biopsy case**. *Internal medicine* 2006, **45**(10):693-695.
146. Inooka G, Ishikawa S, Saito T, Saito K, Kamoshida T, Kuzuya T: **An autopsy case of intravascular lymphomatosis (neoplastic angioendotheliomatosis) accompanied by high fever, hypertension and without focal sign**. *Internal medicine* 1992, **31**(5):666-670.
147. Ip M, Chan KW, Chan IK: **Systemic inflammatory response syndrome in intravascular lymphomatosis**. *Intensive care medicine* 1997, **23**(7):783-786.
148. Ishida M, Hodohara K, Yoshida T, Okabe H: **Intravascular large B-cell lymphoma colonizing in senile hemangioma: a case report and proposal of possible diagnostic strategy for intravascular lymphoma**. *Pathology international* 2011, **61**(9):555-557.
149. Ishiguro T, Takayanagi N, Yanagisawa T, Kagiyama N, Saito H, Sugita Y, Kojima M: **Pulmonary microvascular cytology can detect tumor cells of intravascular lymphoma**. *Internal medicine* 2009, **48**(16):1425-1428.
150. Ishii W, Ito S, Kondo Y, Tsuboi H, Mamura M, Goto D, Matsumoto I, Tsutsumi A, Sumida T, Okoshi Y *et al*: **Intravascular large B-cell lymphoma with acute abdomen as a presenting symptom in a patient with systemic lupus erythematosus**. *Journal of clinical oncology : official journal of the American Society of Clinical Oncology* 2008, **26**(9):1553-1555.

151. Ishiko J, Mizuki M, Yasumi M, Ujiie H, Nakamichi I, Aozasa K, Kanakura Y: **An indolent subtype of "intravascular lymphoma": A case with a 3-year history of LDH elevation**. *Leukemia & lymphoma* 2007, **48**(9):1872-1874.
152. Isimbaldi G, Corral L, Songia S, Valente MG, De Bianchi S, Biondi A: **An unusual presentation of a case of T cell angiotropic (intravascular) lymphoma**. *Leukemia : official journal of the Leukemia Society of America, Leukemia Research Fund, UK* 2000, **14**(12):2321-2322.
153. Ito M, Kim Y, Choi JW, Ozawa H, Fujino M: **Prevalence of intravascular large B-cell lymphoma with bone marrow involvement at initial presentation**. *International journal of hematology* 2003, **77**(2):159-163.
154. Jalkanen S, Aho R, Kallajoki M, Ekfors T, Nortamo P, Gahmberg C, Duijvestijn A, Kalimo H: **Lymphocyte homing receptors and adhesion molecules in intravascular malignant lymphomatosis**. *International journal of cancer Journal international du cancer* 1989, **44**(5):777-782.
155. Jang HJ, Lee KS, Han J: **Intravascular lymphomatosis of the lung: radiologic findings**. *Journal of computer assisted tomography* 1998, **22**(3):427-429.
156. Jardin F, Callonnec F, Contentin N, Picquenot JM, Gueit I, Heron F, Bastard C, Tilly H: **Intravascular large B-Cell lymphoma with bone marrow involvement and superior sagittal sinus thrombosis: report of a case successfully treated with a CHOP/rituximab combination regimen**. *Clinical lymphoma* 2005, **6**(1):46-49.
157. Jiang QL, Pytel P, Rowin J: **Disseminated intravascular large-cell lymphoma with initial presentation mimicking Guillain-Barre syndrome**. *Muscle & nerve* 2010, **42**(1):133-136.
158. Jitpratoom P, Yuckpan P, Sitthinamsuwan P, Chotinaiwattarakul W, Chinthammitr Y: **Progressive multifocal cerebral infarction from intravascular large B cell lymphoma presenting in a man: a case report**. *Journal of medical case reports* 2011, **5**:24.
159. Jones JM, Ceballos R: **Neoplastic angioendotheliomatosis and prostatic carcinoma coexisting in a patient. A case report**. *The Alabama journal of medical sciences* 1986, **23**(3):318-321.
160. Kaku N, Seki M, Doi S, Hayashi T, Imanishi D, Imamura Y, Kurihara S, Miyazaki T, Izumikawa K, Kakeya H *et al*: **A case of intravascular large B-cell lymphoma (IVLBCL) with no abnormal findings on chest computed tomography diagnosed by random transbronchial lung biopsy**. *Internal medicine* 2010, **49**(24):2697-2701.
161. Kakumitsu H, Higuchi M, Tanaka K, Shibuya T: **Nephrotic syndrome in a patient with intravascular lymphomatosis**. *Internal medicine* 2003, **42**(1):98-101.
162. Kamath NV, Gilliam AC, Nihal M, Spiro TP, Wood GS: **Primary cutaneous large B-cell lymphoma of the leg relapsing as cutaneous intravascular large B-cell lymphoma**. *Archives of dermatology* 2001, **137**(12):1657-1658.
163. Kameoka Y, Takahashi N, Komatsuda A, Tagawa H, Hamai K, Hirokawa M, Wakui H, Ichinohasama R, Sawada K: **Kidney-limited intravascular large B cell lymphoma: a distinct variant of IVLBCL?** *International journal of hematology* 2009, **89**(4):533-537.

164. Kameoka Y, Takahashi N, Tagawa H, Fujishima N, Yoshioka T, Saito H, Hirokawa M, Ichinohasama R, Sawada K: **A case of intravascular large B-cell lymphoma of the cutaneous variant: the first case in Asia**. *International journal of hematology* 2010, **91**(1):146-148.
165. Kamesaki H, Matsui Y, Ohno Y, Amano H, Imanaka T, Takahashi Y, Kobashi Y, Kawakami K, Kita K: **Angiotropic lymphoma with histologic features of neoplastic angioendotheliomatosis presenting with predominant respiratory and hematologic manifestations. Report of a case and review of the literature [corrected]**. *American journal of clinical pathology* 1990, **94**(6):768-772.
166. Kanda M, Suzumiya J, Ohshima K, Haraoka S, Nakamura N, Abe M, Tamura K, Kikuchi M: **Analysis of the immunoglobulin heavy chain gene variable region of intravascular large B-cell lymphoma**. *Virchows Archiv : an international journal of pathology* 2001, **439**(4):540-546.
167. Kanda M, Suzumiya J, Ohshima K, Tamura K, Kikuchi M: **Intravascular large cell lymphoma: clinicopathological, immuno-histochemical and molecular genetic studies**. *Leukemia & lymphoma* 1999, **34**(5-6):569-580.
168. Kanno M, Nakamura S, Kawahara M, Ueno S, Nakamine H, Matsumoto M, Fujimura Y: **Chemotherapy-resistant intravascular lymphoma accompanied by ADAMTS13 inhibitor successfully treated with rituximab**. *International journal of hematology* 2008, **88**(3):345-347.
169. Kano M, Yoshida J, Hashizume Y, Sugita K: **Factor VIII can be positive in a special type of malignant lymphoma, intravascular malignant lymphomatosis: an immunohistochemical investigation**. *Noshuyo byori = Brain tumor pathology* 1994, **11**(2):135-141.
170. Kao NL, Broy S, Tillawi I: **Malignant angioendotheliomatosis mimicking systemic necrotizing vasculitis**. *The Journal of rheumatology* 1992, **19**(7):1133-1135.
171. Kasuya A, Hashizume H, Takigawa M: **Early diagnosis of recurrent diffuse large B-cell lymphoma showing intravascular lymphoma by random skin biopsy**. *The Journal of dermatology* 2011, **38**(6):571-574.
172. Katalinic D, Valkovic T, Lucin K, Rudez J: **Intravascular lymphoma and thyroid gland**. *Collegium antropologicum* 2006, **30**(1):239-241.
173. Kato M, Ohshima K, Mizuno M, Kyogoku M, Hashikawa K, Tokura Y, Miyachi Y, Kabashima K: **Analysis of CXCL9 and CXCR3 expression in a case of intravascular large B-cell lymphoma**. *Journal of the American Academy of Dermatology* 2009, **61**(5):888-891.
174. Kauh YC, McFarland JP, Carnabuci GG, Luscombe HA: **Malignant proliferating angioendotheliomatosis**. *Archives of dermatology* 1980, **116**(7):803-806.
175. Kawahara M, Kanno M, Matsumoto M, Nakamura S, Fujimura Y, Ueno S: **Diffuse neurodeficits in intravascular lymphomatosis with ADAMTS13 inhibitor**. *Neurology* 2004, **63**(9):1731-1733.
176. Kawamura T, Sando Y, Tajima S, Hosono T, Sato M, Maeno Y, Maeno T, Suga T, Kurabayashi M, Nagai R: **Pulmonary intravascular lymphoma complicated with Pneumocystis carinii pneumonia: a case report**. *Japanese journal of clinical oncology* 2001, **31**(7):333-336.

177. Kaya H, Yoshida T: **A case of intravascular lymphoma complicated with Fournier's syndrome due to multidrug-resistant Pseudomonas aeruginosa**. *Journal of clinical and experimental hematopathology : JCEH* 2011, **51**(2):115-118.
178. Kayano H, Katayama I: **Primary hepatic lymphoma presenting as intravascular lymphomatosis**. *Archives of pathology & laboratory medicine* 1990, **114**(6):580-584.
179. Keahey TM, Guerry Dt, Tuthill RJ, Bondi EE: **Malignant angioendotheliomatosis proliferans treated with doxorubicin**. *Archives of dermatology* 1982, **118**(7):512-514.
180. Kenez J, Barsi P, Majtenyi K, Molnar B, Kocher I, Stangl E, Komoly S: **Can intravascular lymphomatosis mimic sinus thrombosis? A case report with 8 months' follow-up and fatal outcome**. *Neuroradiology* 2000, **42**(6):436-440.
181. Khoury H, Dalal BI, Nantel SH: **Intravascular lymphoma presenting with bone marrow involvement and leukemic phase**. *Leukemia & lymphoma* 2003, **44**(6):1043-1047.
182. Khoury H, Lestou VS, Gascoyne RD, Bruyere H, Li CH, Nantel SH, Dalal BI, Naiman SC, Horsman DE: **Multicolor karyotyping and clinicopathological analysis of three intravascular lymphoma cases**. *Modern pathology : an official journal of the United States and Canadian Academy of Pathology, Inc* 2003, **16**(7):716-724.
183. Kidson-Gerber G, Bosco A, Maccallum S, Dunkley S: **Two cases of intravascular lymphoma: highlighting the diagnostic difficulties in pyrexia of unknown origin**. *Internal medicine journal* 2005, **35**(9):569-570.
184. Kinoshita T, Sugihara S, Matusue E, Nomura T, Ametani M, Ohama E, Ogawa T: **Intravascular malignant lymphomatosis: diffusion-weighted magnetic resonance imaging characteristics**. *Acta radiologica* 2005, **46**(3):246-249.
185. Kitanaka A, Kubota Y, Imataki O, Ohnishi H, Fukumoto T, Kurokohchi K, Tanaka T: **Intravascular large B-cell lymphoma with FDG accumulation in the lung lacking CT/(67)gallium scintigraphy abnormality**. *Hematological oncology* 2009, **27**(1):46-49.
186. Kivity S, Shalmon B, Sidi Y: **Guillain-Barre syndrome: an unusual presentation of intravascular lymphoma**. *The Israel Medical Association journal : IMAJ* 2006, **8**(2):137-138.
187. Kiyohara T, Kumakiri M, Kobayashi H, Shimizu T, Ohkawara A, Ohnuki M: **A case of intravascular large B-cell lymphoma mimicking erythema nodosum: the importance of multiple skin biopsies**. *Journal of cutaneous pathology* 2000, **27**(8):413-418.
188. Ko YH, Han JH, Go JH, Kim DS, Kwon OJ, Yang WI, Shin DH, Ree HJ: **Intravascular lymphomatosis: a clinicopathological study of two cases presenting as an interstitial lung disease**. *Histopathology* 1997, **31**(6):555-562.
189. Kobayashi T, Ohno H: **Intravascular large B-cell lymphoma associated with t(14;19)(q32;q13) translocation**. *Internal medicine* 2011, **50**(18):2007-2010.
190. Kobrich U, Falk S, Karhoff M, Middeke B, Anselstetter V, Stutte HJ: **Primary large cell lymphoma of the splenic sinuses: a variant of angiotropic B-cell**

lymphoma (neoplastic angioendotheliomatosis)? *Human pathology* 1992, **23**(10):1184-1187.
191. Koizumi M, Nishimura M, Yokota A, Munekata S, Kobayashi T, Saito Y: **Successful treatment of intravascular malignant lymphomatosis with high-dose chemotherapy and autologous peripheral blood stem cell transplantation**. *Bone marrow transplantation* 2001, **27**(10):1101-1103.
192. Kong YY, Dai B, Sheng WQ, Yang WT, Wang CF, Kong JC, Shi DR: **Intravascular large B-cell lymphoma with cutaneous manifestations: a clinicopathologic, immunophenotypic and molecular study of three cases**. *Journal of cutaneous pathology* 2009, **36**(8):865-870.
193. Kotake T, Kosugi S, Takimoto T, Nakata S, Shiga J, Nagate Y, Nakagawa T, Take H, Katagiri S: **Intravascular large B-cell lymphoma presenting pulmonary arterial hypertension as an initial manifestation**. *Internal medicine* 2010, **49**(1):51-54.
194. Koyama T, O'Uchi T, Matsue K: **Neurolymphomatosis involving the trigeminal nerve and deep peroneal nerve in a patient with relapsed intravascular large B-cell lymphoma**. *European journal of haematology* 2010, **85**(3):275-276.
195. Kraus MD, Jones D, Bartlett NL: **Intravascular lymphoma associated with endocrine dysfunction: a report of four cases and a review of the literature**. *The American journal of medicine* 1999, **107**(2):169-176.
196. Kreiss Y, Schwartz E, Kaminski N, Raanani P, Schiby G, Zvas Z, Sidi Y: **Unique pulmonary presentation of intravascular large cell lymphoma**. *Respiratory medicine* 1998, **92**(8):1087-1089.
197. Krishnan C, Moline S, Anders K, Warnke RA: **Intravascular ALK-positive anaplastic large-cell lymphoma mimicking inflammatory breast carcinoma**. *Journal of clinical oncology : official journal of the American Society of Clinical Oncology* 2009, **27**(15):2563-2565.
198. Krokowski M, Sellmann L, Feller AC: **Intravascular large B-cell lymphoma within a subcutaneous cavernous haemangioma**. *British journal of haematology* 2010, **151**(1):2.
199. Kumar N, Keegan BM, Rodriguez FJ, Hammack JE, Kantarci OH: **Intravascular lymphoma presenting as a longitudinally-extensive myelitis: diagnostic challenges and etiologic clues**. *Journal of the neurological sciences* 2011, **303**(1-2):146-149.
200. Kuo TT, Chen MJ, Kuo MC: **Cutaneous intravascular NK-cell lymphoma: report of a rare variant associated with Epstein-Barr virus**. *The American journal of surgical pathology* 2006, **30**(9):1197-1201.
201. Kurrein F: **Systemic angioendotheliomatosis with metastases**. *Journal of clinical pathology* 1976, **29**(4):347-353.
202. Kusaba T, Hatta T, Tanda S, Kameyama H, Tamagaki K, Okigaki M, Inaba T, Shimazaki C, Sasaki S: **Histological analysis on adhesive molecules of renal intravascular large B cell lymphoma treated with CHOP chemotherapy and rituximab**. *Clinical nephrology* 2006, **65**(3):222-226.
203. Kuvliev E, Glamour T, Shekar R, West BC: **Angiotropic large cell lymphoma presenting as fever of unknown origin**. *The American journal of the medical sciences* 1999, **317**(4):266-268.

204. Kuwabara H: **Intravascular lymphomatosis presenting as bilateral adrenal enlargement and insufficiency**. *Acta cytologica* 1999, **43**(5):975-976.
205. Kuwahara K, Fukata J, Kamio M, Mochizuki T, Tsuchiya A, Tanaka S: **Angiotropic large cell lymphoma which infiltrated to the adrenal glands presenting as reversible adrenal insufficiency**. *Internal medicine* 1998, **37**(1):73-76.
206. Lacomis D, Smith TW, Long RR: **Angiotropic lymphoma (intravascular large cell lymphoma) presenting with cauda equina syndrome**. *Clinical neurology and neurosurgery* 1992, **94**(4):311-315.
207. Langan SM, O'Briain S, Barnes L: **Dermatomyositis associated with angiotropic lymphoma**. *Clinical and experimental dermatology* 2003, **28**(6):597-599.
208. Lannoo L, Smets S, Steenkiste E, Delforge M, Moerman P, Stroobants S, Knockaert D, Amant F: **Intravascular large B-cell lymphoma of the uterus presenting as fever of unknown origin (FUO) and revealed by FDG-PET**. *Acta clinica Belgica* 2007, **62**(3):187-190.
209. Lapkuviene O, Forchetti D, Roepke JE: **Unusual sites of involvement by hematologic malignancies. Case 1. Intravascular large B-cell lymphoma presenting with CNS symptoms**. *Journal of clinical oncology : official journal of the American Society of Clinical Oncology* 2001, **19**(19):3988-3991.
210. Laurino L, Melato M: **Malignant angioendotheliomatosis (Angiotropic lymphoma) of the gallbladder**. *Virchows Archiv A, Pathological anatomy and histopathology* 1990, **417**(3):243-246.
211. Le EN, Gerstenblith MR, Gelber AC, Manno RL, Ranasinghe PD, Sweren RJ, McGirt LY: **The use of blind skin biopsy in the diagnosis of intravascular B-cell lymphoma**. *Journal of the American Academy of Dermatology* 2008, **59**(1):148-151.
212. Le K, Lim A, Bullpitt P, Wood G: **Intravascular B-cell lymphoma diagnosed by skin biopsy**. *The Australasian journal of dermatology* 2005, **46**(4):261-265.
213. Lee BS, Frankfort BJ, Eberhart CG, Weinberg RS: **Diagnosis of intravascular lymphoma by a novel biopsy site**. *Ophthalmology* 2011, **118**(3):586-590.
214. Lee HB, Pulido JS, Buettner H, Salomao D, Zent CS, Link TP: **Intravascular B-cell lymphoma (angiotropic lymphoma) with choroidal involvement**. *Archives of ophthalmology* 2006, **124**(9):1357-1359.
215. Legeais M, Gallas S, Cottier JP, Herbreteau D: **Paraplegia and sensory deficit caused by angiotropic large cell lymphoma**. *AJNR American journal of neuroradiology* 2004, **25**(10):1831-1835.
216. Levin KH, Lutz G: **Angiotropic large-cell lymphoma with peripheral nerve and skeletal muscle involvement: early diagnosis and treatment**. *Neurology* 1996, **47**(4):1009-1011.
217. Liao JB, Hsieh PP, Hwang YC, Lin SL, Wu CS: **Cutaneous intravascular natural killer-cell lymphoma: a rare case and review of the literature**. *Acta dermato-venereologica* 2011, **91**(4):472-473.
218. Lie JT: **Malignant angioendotheliomatosis (intravascular lymphomatosis) clinically simulating primary angiitis of the central nervous system**. *Arthritis and rheumatism* 1992, **35**(7):831-834.

219. Liew CL, Shyu WC, Tsao WL, Li H: **Intravascular lymphomatosis mimicks a cerebral demyelinating disorder**. *Acta neurologica Taiwanica* 2006, **15**(4):264-268.
220. Lim HW, Anderson HM: **Angioendotheliomatosis associated with histiocytic lymphoma. Response to systemic chemotherapy**. *Journal of the American Academy of Dermatology* 1985, **13**(5 Pt 2):903-908.
221. Liszka U, Drlicek M, Hitzenberger P, Machacek E, Mayer H, Stockhammer G, Grisold W: **Intravascular lymphomatosis: a clinicopathological study of three cases**. *Journal of cancer research and clinical oncology* 1994, **120**(3):164-168.
222. Liu H, Koyanagi I, Chiba H, Wanibuchi M, Honmou O, Yamaki T, Houkin K: **Spinal cord infarct as the initial clinical presentation of intravascular malignant lymphomatosis**. *Journal of clinical neuroscience : official journal of the Neurosurgical Society of Australasia* 2009, **16**(4):570-573.
223. Lopez-Gil F, Roura M, Umbert I, Umbert P: **Malignant proliferative angioendotheliomatosis or angiotropic lymphoma associated with a soft-tissue lymphoma**. *Journal of the American Academy of Dermatology* 1992, **26**(1):101-104.
224. Lozsadi DA, Wieshmann U, Enevoldson TP: **Neurological presentation of intravascular lymphoma: report of two cases and discussion of diagnostic challenges**. *European journal of neurology : the official journal of the European Federation of Neurological Societies* 2005, **12**(9):710-714.
225. Lu PH, Kuo TT, Yu KH, Lin TL, Chang SL, Yang CH: **Intravascular large B-cell lymphoma presenting in subcutaneous fat tissue and simulating panniculitis clinically**. *International journal of dermatology* 2009, **48**(12):1349-1352.
226. Lui PC, Wong GK, Poon WS, Tse GM: **Intravascular lymphomatosis**. *Journal of clinical pathology* 2003, **56**(6):468-470.
227. Ma X, Liu H: **Intravascular large B-cell lymphoma originating in the left epididymis**. *Annals of hematology* 2011, **90**(1):107-108.
228. Madara J, Shane J, Scarlato M: **Systemic endotheliomatosis: a case report**. *Journal of clinical pathology* 1975, **28**(6):476-482.
229. Maejima H, Tanei R, Morioka T, Miyakoshi S: **Haemophagocytosis-related intravascular large B-cell lymphoma associated with skin eruption**. *Acta dermato-venereologica* 2011, **91**(3):339-340.
230. Maisey NR, Waters JS, Collins D, Schofield J, Hill ME: **A rare case of intravascular lymphoma diagnosed on bone marrow trephine**. *Leukemia & lymphoma* 2003, **44**(11):1997-2000.
231. Malicki DM, Suh YK, Fuller GN, Shin SS: **Angiotropic (intravascular) large cell lymphoma of T-cell phenotype presenting as acute appendicitis in a patient with acquired immunodeficiency syndrome**. *Archives of pathology & laboratory medicine* 1999, **123**(4):335-337.
232. Manckoundia P, Rigaud-Royer I, Berthier S, Popitean L, Bonnotte B, Justrabo E, Pfitzenmeyer P, Lorcerie B: **Intravascular malignant lymphomatosis diagnosed on a muscular biopsy: a case report**. *European journal of internal medicine* 2004, **15**(3):190-192.

233. Mandal AK, Savvidou L, Slater RM, Cockett W, Wiggins J, Missouris CG: **Angiotropic lymphoma: Associated chromosomal abnormalities**. *European journal of internal medicine* 2007, **18**(5):432-434.
234. Marini-Bettolo C, Lane R, Charles P, Naresh K, Nicholas R, Singh P, Cohen A, Mackie P, Roncaroli F: **Myopathy secondary to intravascular large B-cell lymphoma**. *Neuromuscular disorders : NMD* 2009, **19**(12):856-859.
235. Martin-Duverneuil N, Mokhtari K, Behin A, Lafitte F, Hoang-Xuan K, Chiras J: **Intravascular malignant lymphomatosis**. *Neuroradiology* 2002, **44**(9):749-754.
236. Martusewicz-Boros M, Wiatr E, Radzikowska E, Roszkowski-Sliz K, Langfort R: **Pulmonary intravascular large B-cell lymphoma as a cause of severe hypoxemia**. *Journal of clinical oncology : official journal of the American Society of Clinical Oncology* 2007, **25**(15):2137-2139.
237. Masaki Y, Dong L, Nakajima A, Iwao H, Miki M, Kurose N, Kinoshita E, Nojima T, Sawaki T, Kawanami T *et al*: **Intravascular large B cell lymphoma: proposed of the strategy for early diagnosis and treatment of patients with rapid deteriorating condition**. *International journal of hematology* 2009, **89**(5):600-610.
238. Massimino M, Giardini R, Cefalo G, Simonetti F, Pollo B, Giombini S, Tesoro-Tess JD, Ponzoni M, Patriarca C: **Intravascular lymphomatosis (IL) in a child mimicking a posterior fossa tumor**. *Journal of neuro-oncology* 2001, **51**(1):47-50.
239. Matsue K, Asada N, Odawara J, Aoki T, Kimura S, Iwama K, Fujiwara H, Yamakura M, Takeuchi M: **Random skin biopsy and bone marrow biopsy for diagnosis of intravascular large B cell lymphoma**. *Annals of hematology* 2011, **90**(4):417-421.
240. Matsue K, Asada N, Takeuchi M, Yamakura M, Kimura S, Odawara J, Aoki T: **A clinicopathological study of 13 cases of intravascular lymphoma: experience in a single institution over a 9-yr period**. *European journal of haematology* 2008, **80**(3):236-244.
241. Matsue K, Takeuchi M, Uryu H, Koseki M, Asada N, Kaneko Y: **Rapid improvement of hypoxemia by the use of rituximab in patients with pulmonary intravascular lymphoma**. *Leukemia & lymphoma* 2007, **48**(1):197-200.
242. Merchant SH, Viswanatha DS, Zumwalt RE, Foucar K: **Epstein-Barr virus-associated intravascular large T-cell lymphoma presenting as acute renal failure in a patient with acquired immune deficiency syndrome**. *Human pathology* 2003, **34**(9):950-954.
243. Mirza A, Torretti D, Tyler W, Pachipala K: **Angiotropic large cell lymphoma presenting as fever of unknown origin**. *American journal of hematology* 2002, **71**(3):234-235.
244. Miura Y, Matsui Y, Sugino N, Nakato Y, Takeda H, Iwai F, Toyooka N, Kaneko H, Watanabe M, Tsudo M: **Intravascular large B-cell lymphoma cells in the bone marrow smear preparation**. *British journal of haematology* 2011, **152**(2):237-238.
245. Miyazaki C, Mukai M, Kawaai Y, Takeda M, Katoh N, Nagano S, Kubo K, Kohno M: **A case of intravascular lymphoma with increased regional cerebral**

blood flow in I-123 IMP single-photon emission CT. *AJNR American journal of neuroradiology* 2004, **25**(4):565-570.
246. Miyoshi I, Kubota T, Saito T, Toi M, Taguchi H: **Intravascular lymphoma presenting with diverse neurologic manifestations**. *Internal medicine* 2006, **45**(2):119-120.
247. Mleczko A, Franke I, Scheinpflug K, Gollnick H, Leverkus M: **Intravascular large B-cell lymphoma: successful therapy with bendamustine and rituximab**. *Acta dermato-venereologica* 2009, **89**(4):425-427.
248. Mock DJ, Jundt JW, Green JB, Speights VO: **Angiotropic lymphoma manifested by fever and painful swollen legs**. *Southern medical journal* 1993, **86**(12):1432-1435.
249. Molina A, Lombard C, Donlon T, Bangs CD, Dorfman RF: **Immunohistochemical and cytogenetic studies indicate that malignant angioendotheliomatosis is a primary intravascular (angiotropic) lymphoma**. *Cancer* 1990, **66**(3):474-479.
250. Monteiro M, Duarte I, Cabecadas J, Orvalho ML: **Intravascular large B-cell lymphoma of the breast**. *Breast* 2005, **14**(1):75-78.
251. Morikawa Y, Tohya K, Kuribayashi K, Saito K: **A case of neoplastic angioendotheliomatosis: angiotropic lymphoma**. *Applied pathology* 1989, **7**(5):322-328.
252. Morimoto K, Ogihara T, Shiomi T, Awaya N: **Intravascular large B-cell lymphoma with preceding syndrome of inappropriate secretion of antidiuretic hormone**. *Internal medicine* 2007, **46**(18):1569-1572.
253. Motegi S, Tamura A, Takeuchi Y, Ishikawa O: **Senile angioma-like eruption: a skin manifestation of intravascular large B cell lymphoma**. *Dermatology* 2004, **209**(2):135-137.
254. Moussouttas M: **Intravascular lymphomatosis presenting as posterior leukoencephalopathy**. *Archives of neurology* 2002, **59**(4):640-641.
255. Mudhar HS, Sethuraman C, Khan MD, Jan SU: **Intracular, pan-uveal intravascular large B-cell lymphoma associated with choroidal infarction and choroidal tri-lineage extramedullary haemtopoiesis**. *Histopathology* 2007, **51**(2):275-279.
256. Muftah S, Xu Z, El Gaddafi W, Moulton R, Burns B, Woulfe J: **Synchronous intravascular large B-cell lymphoma within meningioma**. *Neuropathology : official journal of the Japanese Society of Neuropathology* 2012, **32**(1):77-81.
257. Murase T, Nakamura S, Kawauchi K, Matsuzaki H, Sakai C, Inaba T, Nasu K, Tashiro K, Suchi T, Saito H: **An Asian variant of intravascular large B-cell lymphoma: clinical, pathological and cytogenetic approaches to diffuse large B-cell lymphoma associated with haemophagocytic syndrome**. *British journal of haematology* 2000, **111**(3):826-834.
258. Murase T, Nakamura S, Tashiro K, Suchi T, Hiraga J, Hayasaki N, Kimura M, Murakami M, Mizoguchi Y, Suzuki T *et al*: **Malignant histiocytosis-like B-cell lymphoma, a distinct pathologic variant of intravascular lymphomatosis: a report of five cases and review of the literature**. *British journal of haematology* 1997, **99**(3):656-664.

259. Murase T, Yamaguchi M, Suzuki R, Okamoto M, Sato Y, Tamaru J, Kojima M, Miura I, Mori N, Yoshino T *et al*: **Intravascular large B-cell lymphoma (IVLBCL): a clinicopathologic study of 96 cases with special reference to the immunophenotypic heterogeneity of CD5**. *Blood* 2007, **109**(2):478-485.
260. Muto G, Takahashi Y, Yamashita H, Mimori A: **A patient with intravascular lymphoma presenting with cerebral infarction and a high serum MPO-ANCA level**. *Modern rheumatology / the Japan Rheumatism Association* 2011, **21**(2):207-210.
261. Nagayama M, Shinohara Y, Sekiyama S, Takahashi W, Takagi S, Yamamoto M, Tanaka S, Inada K: **Intravascular malignant lymphomatosis manifesting clinically as bilateral sudden hearing loss and cytomegalovirus encephalitis**. *Neurology* 1994, **44**(8):1518-1520.
262. Nakahara T, Saito T, Muroi A, Sugiura Y, Ogata M, Sugiyama Y, Yamamoto T: **Intravascular lymphomatosis presenting as an ascending cauda equina: conus medullaris syndrome: remission after biweekly CHOP therapy**. *Journal of neurology, neurosurgery, and psychiatry* 1999, **67**(3):403-406.
263. Nakajima S, Ohshima K, Kyogoku M, Miyachi Y, Kabashima K: **A case of intravascular large B-cell lymphoma with atypical clinical manifestations and analysis of CXCL12 and CXCR4 expression**. *Archives of dermatology* 2010, **146**(6):686-687.
264. Nakamichi I, Shimazu K, Ikeda J, Yamauchi A, Ishiko J, Mizuki M, Kanakura Y, Aozasa K: **Intravascular lymphomatosis initially suspected from uterine cytology: a case report**. *Acta cytologica* 2009, **53**(2):198-200.
265. Nakamichi N, Fukuhara S, Aozasa K, Morii E: **NK-cell intravascular lymphomatosis--a mini-review**. *European journal of haematology* 2008, **81**(1):1-7.
266. Nakamura T, Watanabe M, Hotchi M, Fujimori N, Mizuno M: **Neoplastic angioendotheliomatosis. Report of two autopsy cases with special reference to the origin of atypical cells**. *Acta pathologica japonica* 1987, **37**(8):1337-1346.
267. Nakamura Y, Nakamagoe K, Kawachi Y, Hosaka A, Mukai H, Chiba S, Otsuka F, Tamaoka A: **Intravascular large B cell lymphoma with neurological symptoms diagnosed on the basis of a senile angioma-like eruption**. *BMJ case reports* 2009, **2009**.
268. Nakanuma Y, Kumabashiri I: **Neoplastic angioendotheliomatosis with multifocal hemorrhagic necrosis of the liver**. *The American journal of gastroenterology* 1988, **83**(10):1180-1182.
269. Nakashima MO, Roy DB, Nagamine M, Roullet MR, Gabriel CA, Sood SL, Bagg A: **Intravascular large B-cell lymphoma: a mimicker of many maladies and a difficult and often delayed diagnosis**. *Journal of clinical oncology : official journal of the American Society of Clinical Oncology* 2011, **29**(6):e138-140.
270. Narimatsu H, Morishita Y, Saito S, Shimada K, Ozeki K, Kohno A, Kato Y, Nagasaka T: **Usefulness of bone marrow aspiration for definite diagnosis of Asian variant of intravascular lymphoma: four autopsied cases**. *Leukemia & lymphoma* 2004, **45**(8):1611-1616.

271. Natali-Sora MG, Lodi M, Corbo M, Hays AP, Nemni R: **Intravascular malignant lymphomatosis with neurological symptoms**. *Journal of neurology* 1996, **243**(2):205-206.
272. Niida T, Isoda K, Miyazaki K, Kanoh S, Kobayashi H, Kobayashi A, Kimura F, Hayashi K, Kusuhara M, Ohsuzu F: **Pulmonary intravascular lymphoma diagnosed by 18-fluorodeoxyglucose positron emission tomography-guided transbronchial lung biopsy in a man with long-term survival: a case report**. *Journal of medical case reports* 2011, **5**:295.
273. Niitsu N, Okamura D, Takahashi N, Tanae K, Hagiwara Y, Kayano H, Bessho M: **Renal intravascular large B-cell lymphoma with early diagnosis by renal biopsy: a case report and review of the literature**. *Leukemia research* 2009, **33**(5):728-730.
274. Nishikawa K, Sekiyama S, Suzuki T, Ito Y, Matsukawa W, Tamai H, Yoshida F, Fukatsu A, Matsuo S, Shigematsu H: **A case of angiotropic large cell lymphoma manifesting nephrotic syndrome and treated successfully with combination chemotherapy**. *Nephron* 1991, **58**(4):479-482.
275. Nixon BK, Kussick SJ, Carlon MJ, Rubin BP: **Intravascular large B-cell lymphoma involving hemangiomas: an unusual presentation of a rare neoplasm**. *Modern pathology : an official journal of the United States and Canadian Academy of Pathology, Inc* 2005, **18**(8):1121-1126.
276. Odawara J, Asada N, Aoki T, Yamakura M, Takeuchi M, Ohuchi T, Matsue K: **18F-Fluorodeoxyglucose positron emission tomography for evaluation of intravascular large B-cell lymphoma**. *British journal of haematology* 2007, **136**(5):684.
277. Oei ME, Kraft GH, Sarnat HB: **Intravascular lymphomatosis**. *Muscle & nerve* 2002, **25**(5):742-746.
278. Ohashi N, Aomatsu M, Mori A, Takahashi M, Shibuya T, Maruyama T, Inoue H, Takegoshi S, Yokoi T, Okuno M: **Intravascular lymphoma with extremely low high-density lipoproteinemia**. *Internal medicine* 2007, **46**(17):1475-1477.
279. Ohno T, Sakamoto T, Mizumoto C, Miyoshi T, Ueda M, Takeoka T, Yamashita K, Hishita T, Hada S: **Leukemic and meningeal relapse of CD5+ intravascular large B-cell lymphoma with down-modulation of CD20 after rituximab therapy**. *International journal of hematology* 2006, **84**(1):74-78.
280. Okada Y, Nakanishi I, Nomura H, Takeda R, Nonomura A, Takekuma K: **Angiotropic B-cell lymphoma with hemophagocytic syndrome**. *Pathology, research and practice* 1994, **190**(7):718-724; discussion 725-717.
281. Okagaki T, Richart RM: **Systemic proliferating angioendotheliomatosis. A case report**. *Obstetrics and gynecology* 1971, **37**(3):377-380.
282. Ormsby A, Prayson RA, Heard R: **Angiotrophic large cell lymphoma mimicking multiple sclerosis associated transverse myelitis**. *Journal of clinical neuroscience : official journal of the Neurosurgical Society of Australasia* 1999, **6**(5):408-410.
283. Ossege LM, Postler E, Pleger B, Muller KM, Malin JP: **Neoplastic cells in the cerebrospinal fluid in intravascular lymphomatosis**. *Journal of neurology* 2000, **247**(8):656-658.

284. Otrakji CL, Voigt W, Amador A, Nadji M, Gregorios JB: **Malignant angioendotheliomatosis--a true lymphoma: a case of intravascular malignant lymphomatosis studied by southern blot hybridization analysis**. *Human pathology* 1988, **19**(4):475-478.
285. Owa M, Koyama J, Asakawa K, Hikita H, Kubo K, Ikeda SI: **Intravascular lymphomatosis presenting as reversible severe pulmonary hypertension**. *International journal of cardiology* 2000, **75**(2-3):283-284.
286. Ozguroglu E, Buyulbabani N, Ozguroglu M, Baykal C: **Generalized telangiectasia as the major manifestation of angiotropic (intravascular) lymphoma**. *The British journal of dermatology* 1997, **137**(3):422-425.
287. Pahk PJ, Todd DJ, Blaha GR, Soukiasian SH, Landmann DS, Craven DE, Tronic BS, Zabar Y, Marx JL: **Intravascular lymphoma masquerading as Vogt-Koyanagi-Harada syndrome**. *Ocular immunology and inflammation* 2008, **16**(3):123-126.
288. Papalas JA, Proia AD, Cummings TJ: **Hematolymphoid malignancies with intraocular intravascular involvement: report of 2 cases**. *Annals of diagnostic pathology* 2011, **15**(4):286-290.
289. Park GH, Kim CH, Chung WK, Won CH, Chang SE, Lee MW, Choi JH, Moon KC: **Primary cutaneous intravascular large B-cell lymphoma treated with combination chemotherapy and complicated by rituximab-induced interstitial lung disease**. *Acta dermato-venereologica* 2010, **90**(3):296-298.
290. Park JH, Lee DY, Ko YH: **Intravascular large B-cell lymphoma of the cutaneous variant in Korea**. *The Journal of dermatology* 2011, **38**(2):160-163.
291. Parrens M, Dubus P, Agape P, Rizcallah E, Marit G, de Mascarel A, Merlio JP: **Intrasinusoidal bone marrow infiltration revealing intravascular lymphomatosis**. *Leukemia & lymphoma* 2000, **37**(1-2):219-223.
292. Passarin MG, Wen PY, Vattemi E, Buffone E, Ghimenton C, Bontempini L, Ottaviani S, Musso AM, Pedersini R: **Intravascular lymphomatosis and intracerebral haemorrhage**. *Neurological sciences : official journal of the Italian Neurological Society and of the Italian Society of Clinical Neurophysiology* 2010, **31**(6):793-797.
293. Pasyk K, Depowski M: **Proliferating systematized angioendotheliomatosis of a 5-month-old infant**. *Archives of dermatology* 1978, **114**(10):1512-1515.
294. Pekic S, Milicevic S, Colovic N, Colovic M, Popovic V: **Intravascular large B-cell lymphoma as a cause of hypopituitarism: gradual and late reversal of hypopituitarism after long-term remission of lymphoma with immunochemotherapy**. *Endocrine* 2008, **34**(1-3):11-16.
295. Pellicone JT, Goldstein HB: **Pulmonary malignant angioendotheliomatosis. Presentation with fever and syndrome of inappropriate antidiuretic hormone**. *Chest* 1990, **98**(5):1292-1294.
296. Perniciaro C, Winkelmann RK, Daoud MS, Su WP: **Malignant angioendotheliomatosis is an angiotropic intravascular lymphoma. Immunohistochemical, ultrastructural, and molecular genetics studies**. *The American Journal of dermatopathology* 1995, **17**(3):242-248.

297. Petroff N, Koger OW, Fleming MG, Fishleder A, Bergfeld WF, Tuthill R, Tubbs R: **Malignant angioendotheliomatosis: an angiotropic lymphoma**. *Journal of the American Academy of Dermatology* 1989, **21**(4 Pt 1):727-733.
298. Piyatanont K, Bamrungrak K, Watcharananan S, Sathapatayavongs B, Chuncharunee S, Larbcharoensub N, Witoonpanich R: **Intravascular B-cell lymphoma presenting with cauda equina syndrome: the role of skin biopsy**. *European journal of dermatology : EJD* 2010, **20**(6):821-822.
299. Ponzoni M, Arrigoni G, Gould VE, Del Curto B, Maggioni M, Scapinello A, Paolino S, Cassisa A, Patriarca C: **Lack of CD 29 (beta1 integrin) and CD 54 (ICAM-1) adhesion molecules in intravascular lymphomatosis**. *Human pathology* 2000, **31**(2):220-226.
300. Prayson RA: **Angiotropic large cell lymphoma: simultaneous peripheral nerve and skeletal muscle involvement**. *Pathology* 1996, **28**(1):25-27.
301. Prayson RA, Segal GH, Stoler MH, Licata AA, Tubbs RR: **Angiotropic large-cell lymphoma in a patient with adrenal insufficiency**. *Archives of pathology & laboratory medicine* 1991, **115**(10):1039-1041.
302. Price DA, Thaker H, James A, Snow MH: **Hypopituitarism in a patient with intravascular lymphomatosis**. *Haematologica* 2002, **87**(11):ECR36.
303. Pusch G, Feher G, Szomor A, Kover F, Gomori E, Illes Z: **Intravascular lymphoma presenting with neurological signs but diagnosed by prostate biopsy: suspicion as a key to early diagnosis**. *European journal of neurology : the official journal of the European Federation of Neurological Societies* 2009, **16**(3):e39-41.
304. Quadri AM, Sylvester S, Verma S, Bareford D, Spychal RT, Jones EL, Ganesan R: **Angiotropic large cell lymphoma presenting as intestinal obstruction**. *Annals of hematology* 2008, **87**(1):67-69.
305. Rahman FPS: **Intravascular lymphoma**. *Blood* 2009, **114**:1140.
306. Raroque HG, Jr., Mandler RN, Griffey MS, Orrison WW, Kornfeld M: **Neoplastic angioendotheliomatosis**. *Archives of neurology* 1990, **47**(8):929-930.
307. Rashid R, Johnson RJ, Morris S, Dickinson H, Czyz J, O'Connor SJ, Owen RG: **Intravascular large B-cell lymphoma associated with a near-tetraploid karyotype, rearrangement of BCL6, and a t(11;14)(q13;q32)**. *Cancer genetics and cytogenetics* 2006, **171**(2):101-104.
308. Remberger K, Nawrath-Koll I, Gokel JM, Haider M: **Systemic angioendotheliomatosis of the lung**. *Pathology, research and practice* 1987, **182**(2):265-274.
309. Rieger E, Soyer HP, Leboit PE, Metze D, Slovak R, Kerl H: **Reactive angioendotheliomatosis or intravascular histiocytosis? An immunohistochemical and ultrastructural study in two cases of intravascular histiocytic cell proliferation**. *The British journal of dermatology* 1999, **140**(3):497-504.
310. Rieger KE, Polidore T, Warnke R, Kim J: **ALK-negative systemic intravascular anaplastic large cell lymphoma presenting in the skin**. *Journal of cutaneous pathology* 2011, **38**(2):216-220.

311. Rose C, Staumont D, Jouet JP: **Successful autologous bone marrow transplantation in intravascular lymphomatosis**. *British journal of haematology* 1999, **105**(1):313-314.
312. Roshal M, Till BG, Fromm JR, Cherian S: **Intravascular large B cell lymphoma presenting in a liver explant**. *Journal of clinical pathology* 2008, **61**(7):877-878.
313. Roussou P, Ilias I, Fotinou M: **Angiotropic lymphoma: a rare hematological malignancy**. *Haematologica* 1998, **83**(4):376.
314. Roux S, Grossin M, De Bandt M, Palazzo E, Vachon F, Kahn MF: **Angiotropic large cell lymphoma with mononeuritis multiplex mimicking systemic vasculitis**. *Journal of neurology, neurosurgery, and psychiatry* 1995, **58**(3):363-366.
315. Rubin MA, Cossman J, Freter CE, Azumi N: **Intravascular large cell lymphoma coexisting within hemangiomas of the skin**. *The American journal of surgical pathology* 1997, **21**(7):860-864.
316. Rubio A, Poole RM, Brara HS, Taylor N, Powers JM: **Motor neuron disease and angiotropic lymphoma**. *Archives of neurology* 1997, **54**(1):92-95.
317. Sajid RM, Qureshi A: **Involvement of bone marrow with intravascular large B-cell lymphoma**. *Hematology/oncology and stem cell therapy* 2010, **3**(1):39-41.
318. Saleh Z, Kurban M, Ghosn S, Awar G, Kibbi AG: **Generalized telangiectasia: a manifestation of intravascular B-cell lymphoma**. *Dermatology* 2008, **217**(4):318-320.
319. Sanchez-Cano D, Callejas-Rubio JL, Vilanova-Mateu A, Gomez-Morales M, Ortego-Centeno N: **Intravascular lymphoma in a patient with systemic lupus erythematosus: a case report**. *Lupus* 2007, **16**(7):525-528.
320. Sangueza O, Hyder DM, Sangueza P: **Intravascular lymphomatosis: report of an unusual case with T cell phenotype occurring in an adolescent male**. *Journal of cutaneous pathology* 1992, **19**(3):226-231.
321. Sanna P, Bertoni F, Roggero E, Quattropani C, Rusca T, Pedrinis E, Monotti R, Mombelli G, Cavalli F, Zucca E: **Angiotropic (intravascular) large cell lymphoma: case report and short discussion of the literature**. *Tumori* 1997, **83**(4):772-775.
322. Satoh S, Yamazaki M, Yahikozawa H, Ichikawa N, Saito H, Hanyuu N, Hata S, Hachyou M: **Intravascular large B cell lymphoma diagnosed by senile angioma biopsy**. *Internal medicine* 2003, **42**(1):117-120.
323. Satti S, Castillo R: **Intravascular B-cell lymphoma**. *Community Oncology* 2005, **2**(1):55-58.
324. Saurel CA, Personett DA, Edenfield BH, Solberg LA, Jiang L, Menke DM, Tun HW: **Molecular analysis of intravascular large B-cell lymphoma with neoangiogenesis**. *British journal of haematology* 2011, **152**(2):234-236.
325. Savard M, Verreault S, Gould PV, Bernier V, Bouchard JP: **Intravascular lymphoma with conus medullaris syndrome followed by encephalopathy**. *The Canadian journal of neurological sciences Le journal canadien des sciences neurologiques* 2008, **35**(3):366-371.
326. Savarese DM, Zavarin M, Smyczynski MS, Rohrer MJ, Hutzler MJ: **Superior vena cava syndrome secondary to an angiotropic large cell lymphoma**. *Cancer* 2000, **89**(12):2515-2520.

327. Sawa N, Ubara Y, Katori H, Hoshino J, Suwabe T, Tagami T, Takemoto F, Miyakoshi S, Taniguchi S, Ohashi K *et al*: **Renal intravascular large B-cell lymphoma localized only within peritubular capillaries. Report of a case**. *Internal medicine* 2007, **46**(10):657-662.
328. Sawamoto A, Narimatsu H, Suzuki T, Kurahashi S, Sugimoto T, Sugiura I: **Long-term remission after autologous peripheral blood stem cell transplantation for relapsed intravascular lymphoma**. *Bone marrow transplantation* 2006, **37**(2):233-234.
329. Schleinitz N, Bernit E, Mazodier K, Charbonnier A, Horchowski N, Andrac-Meyer L, Veit V, Harle JR: **Two cases of intravascular lymphomatosis disclosing with hypopituitarism**. *Haematologica* 2002, **87**(6):ECR21.
330. Schonfeld SM, Pinto RS, Aleksic S, Pearson J: **Cerebral angioendotheliomatosis: a report of two cases and review of the literature**. *AJNR American journal of neuroradiology* 1985, **6**(3):437-441.
331. Schwarz S, Zoubaa S, Knauth M, Sommer C, Storch-Hagenlocher B: **Intravascular lymphomatosis presenting with a conus medullaris syndrome mimicking disseminated encephalomyelitis**. *Neuro-oncology* 2002, **4**(3):187-191.
332. Scott PW, Silvers DN, Helwig EB: **Proliferating angioendotheliomatosis**. *Archives of pathology* 1975, **99**(6):323-326.
333. Seki K, Miyakoshi S, Lee GH, Matsushita H, Mutoh Y, Nakase K, Ida M, Taniguchi H: **Prostatic acid phosphatase is a possible tumor marker for intravascular large B-cell lymphoma**. *The American journal of surgical pathology* 2004, **28**(10):1384-1388.
334. Sengupta S, Pedersen NP, Davis JE, Rojas R, Reddy H, Kasper E, Greenstein P, Wong ET: **Illusion of stroke: intravascular lymphomatosis**. *Reviews in neurological diseases* 2011, **8**(3-4):e107-113.
335. Sepandj F, Gupta R, Foyle A: **Renal manifestations of angiotrophic lymphoma: clinicopathological features**. *Nephrology, dialysis, transplantation : official publication of the European Dialysis and Transplant Association - European Renal Association* 1997, **12**(1):190-194.
336. Sepp N, Schuler G, Romani N, Geissler D, Gattringer C, Burg G, Bartram CR, Fritsch P: **"Intravascular lymphomatosis" (angioendotheliomatosis): evidence for a T-cell origin in two cases**. *Human pathology* 1990, **21**(10):1051-1058.
337. Setoyama M, Mizoguchi S, Orikawa T, Tashiro M: **A case of intravascular malignant lymphomatosis (angiotropic large-cell lymphoma) presenting memory T cell phenotype and its expression of adhesion molecules**. *The Journal of dermatology* 1992, **19**(5):263-269.
338. Shan SJ, Chen J, Geng SL, Hong Y, Xu H, Guo Y, Wei H, Zhai M, Chen HD: **Successful treatment of cutaneous intravascular large B-cell lymphoma with fludarabine phosphate**. *European journal of dermatology : EJD* 2010, **20**(3):408-409.
339. Sheibani K, Battifora H, Winberg CD, Burke JS, Ben-Ezra J, Ellinger GM, Quigley NJ, Fernandez BB, Morrow D, Rappaport H: **Further evidence that "malignant angioendotheliomatosis" is an angiotropic large-cell lymphoma**. *The New England journal of medicine* 1986, **314**(15):943-948.

340. Shen Q, Duan X, Feng W, Nguyen N, Lapus A, Brown RE, Chen L: **Intravascular large B-cell lymphoma: report of three cases and analysis of the mTOR pathway**. *International journal of clinical and experimental pathology* 2011, **4**(8):782-790.
341. Shimada K, Kosugi H, Narimatsu H, Shimada S, Suzuki T, Ito M, Kinoshita T, Mori N, Naoe T: **Sustained remission after rituximab-containing chemotherapy for intravascular large B-cell lymphoma**. *Journal of clinical and experimental hematopathology : JCEH* 2008, **48**(1):25-28.
342. Shimada K, Kosugi H, Shimada S, Narimatsu H, Koyama Y, Suzuki N, Yuge M, Nishibori H, Iwata Y, Nakamura S *et al*: **Evaluation of organ involvement in intravascular large B-cell lymphoma by 18F-fluorodeoxyglucose positron emission tomography**. *International journal of hematology* 2008, **88**(2):149-153.
343. Shimada K, Murase T, Matsue K, Okamoto M, Ichikawa N, Tsukamoto N, Niitsu N, Miwa H, Asaoku H, Kosugi H *et al*: **Central nervous system involvement in intravascular large B-cell lymphoma: a retrospective analysis of 109 patients**. *Cancer science* 2010, **101**(6):1480-1486.
344. Shimizu I, Ichikawa N, Yotsumoto M, Sumi M, Ueno M, Kobayashi H: **Asian variant of intravascular lymphoma: aspects of diagnosis and the role of rituximab**. *Internal medicine* 2007, **46**(17):1381-1386.
345. Shimokawa I, Higami Y, Sakai H, Moriuchi Y, Murase K, Ikeda T: **Intravascular malignant lymphomatosis: a case of T-cell lymphoma probably associated with human T-cell lymphotropic virus**. *Human pathology* 1991, **22**(2):200-202.
346. Shiraki K, Sugimoto K, Deguchi M, Ito N, Masuda C, Takei Y: **Hepatic intravascular large B cell lymphoma**. *Internal medicine* 2007, **46**(20):1761-1762.
347. Sill H, Hofler G, Kaufmann P, Horina J, Spuller E, Kleinert R, Beham-Schmid C: **Angiotropic large cell lymphoma presenting as thrombotic microangiopathy (thrombotic thrombocytopenic purpura)**. *Cancer* 1995, **75**(5):1167-1170.
348. Sips GJ, Amory CF, Delman BN, Kleinman GM, Lipsey LR, Tuhrim S: **Intravascular lymphomatosis of the brain in a patient with myelodysplastic syndrome**. *Nature reviews Neurology* 2009, **5**(5):288-292.
349. Sleater JP, Segal GH, Scott MD, Masih AS: **Intravascular (angiotropic) large cell lymphoma: determination of monoclonality by polymerase chain reaction on paraffin-embedded tissues**. *Modern pathology : an official journal of the United States and Canadian Academy of Pathology, Inc* 1994, **7**(5):593-598.
350. Smadja D, Mas JL, Fallet-Bianco C, Meyniard O, Sicard D, de Recondo J, Rondot P: **Intravascular lymphomatosis (neoplastic angioendotheliosis) of the central nervous system: case report and literature review**. *Journal of neuro-oncology* 1991, **11**(2):171-180.
351. Smith ME, Stamatakos MD, Neuhauser TS: **Intravascular lymphomatosis presenting within angiolipomas**. *Annals of diagnostic pathology* 2001, **5**(2):103-106.

352. Snowden JA, Angel CA, Winfield DA, Pringle JH, West KP: **Angiotropic lymphoma: report of a case with histiocytic features**. *Journal of clinical pathology* 1997, **50**(1):67-70.
353. Snyder LS, Harmon KR, Estensen RD: **Intravascular lymphomatosis (malignant angioendotheliomatosis) presenting as pulmonary hypertension**. *Chest* 1989, **96**(5):1199-1200.
354. Sokol DK, Azzarelli B, Smith RR, Kassing LC, Roos KL, Pascuzzi RM, Blinkhorn RJ, Jr.: **Primary intravascular lymphomatosis associated with Mycobacterium marinum**. *Journal of neuroimaging : official journal of the American Society of Neuroimaging* 1998, **8**(1):47-49.
355. Song DE, Lee MW, Ryu MH, Kang DW, Kim SJ, Huh J: **Intravascular large cell lymphoma of the natural killer cell type**. *Journal of clinical oncology : official journal of the American Society of Clinical Oncology* 2007, **25**(10):1279-1282.
356. Song DK, Boulis NM, McKeever PE, Quint DJ: **Angiotropic large cell lymphoma with imaging characteristics of CNS vasculitis**. *AJNR American journal of neuroradiology* 2002, **23**(2):239-242.
357. Souza CA, Quan K, Seely J, Kravcik S, Burns B: **Pulmonary intravascular lymphoma**. *Journal of thoracic imaging* 2009, **24**(3):231-233.
358. Srivatsa S, Sharma J, Logani S: **Intravascular lymphoma: an unusual diagnostic outcome of an incidentally detected adrenal mass**. *Endocrine practice : official journal of the American College of Endocrinology and the American Association of Clinical Endocrinologists* 2008, **14**(7):884-888.
359. Stahl RL, Chan W, Duncan A, Corley CC, Jr.: **Malignant angioendotheliomatosis presenting as disseminated intravascular coagulopathy**. *Cancer* 1991, **68**(10):2319-2323.
360. Stroup RM, Sheibani K, Moncada A, Purdy LJ, Battifora H: **Angiotropic (intravascular) large cell lymphoma. A clinicopathologic study of seven cases with unique clinical presentations**. *Cancer* 1990, **66**(8):1781-1788.
361. Suarez-Vilela D, Izquierdo-Garcia FM, Ramos-Ortega F: **Intravascular lymphomatosis in T-hepatosplenic lymphoma**. *American journal of clinical pathology* 2002, **117**(4):662-663.
362. Sugimoto KJ, Mori KL, Oshimi K: **Intravascular large B-cell lymphoma**. *American journal of hematology* 2004, **76**(3):291-292.
363. Suh CH, Kim SK, Shin DH, Chung KY, Kim SK: **Intravascular lymphomatosis of the T cell type presenting as interstitial lung disease--a case report**. *Journal of Korean medical science* 1997, **12**(5):457-460.
364. Sukpanichnant S, Visuthisakchai S: **Intravascular lymphomatosis: a study of 20 cases in Thailand and a review of the literature**. *Clinical lymphoma & myeloma* 2006, **6**(4):319-328.
365. Sumer M, Ozon AO, Bakar B, Cila A, Ruacan S: **Intravascular lymphoma masquerading as multiembolic stroke developing after coronary artery by-pass surgery**. *The neurologist* 2009, **15**(2):98-101.
366. Sur M, Ross C, Moens F, Daya D: **Intravascular large B-cell lymphoma of the uterus: a diagnostic challenge**. *International journal of gynecological pathology*

: official journal of the International Society of Gynecological Pathologists 2005, **24**(2):201-203.
367. Suzuki S, Koizumi Y: **Angiotropic lymphoma diagnosed by muscle biopsy**. *Internal medicine* 1997, **36**(4):304-307.
368. Suzumiya J, Ohshima K, Kanda M, Kato A, Shuuda K, Kimura N, Tamura K, Kikuchi M: **Intravascular large cell lymphoma associated with hypoalbuminemia**. *Leukemia & lymphoma* 1998, **32**(1-2):179-182.
369. Svajdler M, Lazurova I, Bohus P, Pal'ko M: **Intravascular variant of diffuse large B-cell lymphoma with combined endocrine involvement**. *Wiener klinische Wochenschrift* 2006, **118**(13-14):422-425.
370. Szots M, Szomor A, Kover F, Pajor L, Komoly S, Kalman E, Gomori E, Illes Z: **Intravascular lymphomatosis of the nervous system**. *Journal of neurology* 2008, **255**(10):1590-1592.
371. Szuba A, Koba M, Rzeszutko M, Rzeszutko W, Dziegiel P, Loboda A, Dulak J, Andrzejak R: **Cutaneous angiogenesis in patient with intravascular lymphoma (IVL): A case report**. *Dermatology online journal* 2010, **16**(8):2.
372. Takacs I, Eros N, Bene I, Bozso F, Tordai L, Karolyi Z, Radvanyi G, Matolcsy A: **Successful treatment of relapse of an intravascular B-cell lymphoma with rituximab-CHOP polychemotherapy**. *Annals of hematology* 2004, **83**(9):608-610.
373. Takahashi E, Kajimoto K, Fukatsu T, Yoshida M, Eimoto T, Nakamura S: **Intravascular large T-cell lymphoma: a case report of CD30-positive and ALK-negative anaplastic type with cytotoxic molecule expression**. *Virchows Archiv : an international journal of pathology* 2005, **447**(6):1000-1006.
374. Takahashi T, Minato M, Tsukuda H, Yoshimoto M, Tsujisaki M: **Successful treatment of intravascular large B-cell lymphoma diagnosed by bone marrow biopsy and FDG-PET scan**. *Internal medicine* 2008, **47**(10):975-979.
375. Takamura K, Nasuhara Y, Mishina T, Matsuda T, Nishimura M, Kawakami Y, Fujita M, Mikuni C, Yamashiro K: **Intravascular lymphomatosis diagnosed by transbronchial lung biopsy**. *The European respiratory journal : official journal of the European Society for Clinical Respiratory Physiology* 1997, **10**(4):955-957.
376. Takizawa S, Shirasugi Y, Nakamura N, Nakagawa S, Tsuchiya T, Ando K, Takagi S: **An atypical form of Asian variant of intravascular large B-cell lymphoma presenting with myelopathy alone for 4 months prior to pancytopenia**. *Internal medicine* 2007, **46**(22):1879-1880.
377. Tan TB, Spaander PJ, Blaisse M, Gerritzen FM: **Angiotropic large cell lymphoma presenting as interstitial lung disease**. *Thorax* 1988, **43**(7):578-579.
378. Terrier B, Aouba A, Vasiliu V, Charlier C, Delarue R, Buzyn A, Hermine O: **Intravascular lymphoma associated with haemophagocytic syndrome: a very rare entity in western countries**. *European journal of haematology* 2005, **75**(4):341-345.
379. Thomson JJ, Walt JV, Ireland R: **Bone marrow trephine biopsy appearances of the intravascular subtype of diffuse large B-cell lymphoma**. *British journal of haematology* 2007, **136**(5):683.

380. Tokura T, Murase T, Toriyama T, Totani Y, Negita M, Akaza K, Ozawa H, Nakagawa A, Nakamura S: **Asian variant of CD5+ intravascular large B-cell lymphoma with splenic infarction**. *Internal medicine* 2003, **42**(1):105-109.
381. Tomasini C, Novelli M, Ponti R, Pippione M, Bernengo MG: **Cutaneous intravascular lymphoma following extravascular lymphoma of the lung**. *Dermatology* 2004, **208**(2):158-163.
382. Torenbeek R, Scheltens P, Strack van Schijindel RJ, Algra PR, Heimans JJ, van der Valk P: **Angiotropic intravascular large-cell lymphoma with massive cerebral extension**. *Journal of neurology, neurosurgery, and psychiatry* 1993, **56**(8):914-916.
383. Tranchida P, Bayerl M, Voelpel MJ, Palutke M: **Testicular ischemia due to intravascular large B-cell lymphoma: a novel presentation in an immunosuppressed individual**. *International journal of surgical pathology* 2003, **11**(4):319-324.
384. Treves TA, Gadoth N, Blumen S, Korczyn AD: **Intravascular malignant lymphomatosis: a cause of subacute dementia**. *Dementia (Basel, Switzerland)* 1995, **6**(5):286-293.
385. Tsukadaira A, Okubo Y, Ogasawara H, Urushibata K, Honda T, Miura I, Kubo K: **Chromosomal aberrations in intravascular lymphomatosis**. *American journal of clinical oncology* 2002, **25**(2):178-181.
386. Tucker TJ, Bardales RH, Miranda RN: **Intravascular lymphomatosis with bone marrow involvement**. *Archives of pathology & laboratory medicine* 1999, **123**(10):952-956.
387. Van Droogenbroeck J, Altintas S, Pollefliet C, Schroyens W, Berneman Z: **Intravascular large B-cell lymphoma or intravascular lymphomatosis: report of a case diagnosed by testicle biopsy**. *Annals of hematology* 2001, **80**(5):316-318.
388. Vandenheede M, Dioh A, de Noordhout AM, Deprez M, Schoenen J: **Intravascular malignant lymphomatosis: report of 2 neurological cases**. *Acta neurologica Belgica* 2002, **102**(2):76-81.
389. Venizelos I, Tamiolakis D, Petrakis G: **High grade primary adrenal intravascular large B-cell lymphoma manifesting as Addison disease**. *Revista espanola de enfermedades digestivas : organo oficial de la Sociedad Espanola de Patologia Digestiva* 2007, **99**(8):471-474.
390. Viali S, Hutchinson DO, Hawkins TE, Croxson MC, Thomas M, Allen JP, Thomas SM, Powell KH: **Presentation of intravascular lymphomatosis as lumbosacral polyradiculopathy**. *Muscle & nerve* 2000, **23**(8):1295-1300.
391. Vieites B, Fraga M, Lopez-Presas E, Pintos E, Garcia-Rivero A, Forteza J: **Detection of t(14;18) translocation in a case of intravascular large B-cell lymphoma: a germinal centre cell origin in a subset of these lymphomas?** *Histopathology* 2005, **46**(4):466-468.
392. Vieren M, Sciot R, Robberecht W: **Intravascular lymphomatosis of the brain: a diagnostic problem**. *Clinical neurology and neurosurgery* 1999, **101**(1):33-36.
393. Vital C, Vital A, Julien J, Rivel J, deMascarel A, Vergier B, Henry P, Barat M, Reiffers J, Broustet A: **Peripheral neuropathies and lymphoma without**

monoclonal gammopathy: a new classification. *Journal of neurology* 1990, **237**(3):177-185.
394. von Kempis J, Kohler G, Herbst EW, Peter HH: **Intravascular lymphoma presenting as symmetric polyarthritis**. *Arthritis and rheumatism* 1998, **41**(6):1126-1130.
395. Vos JM, Bordbar A, Vet RJ, Pals ST, Kater AP: **Waxing and waning intravascular large cell lymphoma with widespread organ infiltration**. *Leukemia & lymphoma* 2011, **52**(4):705-708.
396. Vougiouklakis T, Mitselou A, Agnantis NJ: **Angiotropic large cell lymphoma**. *Journal of experimental & clinical cancer research : CR* 2004, **23**(2):345-348.
397. Vrindavanam N, Hamadani M, Steele B, Awan F, Suster S, Benson DM, Jr.: **Dramatic response to single-agent rituximab in a patient with intravascular lymphoma**. *American journal of hematology* 2007, **82**(12):1120-1121.
398. Wach M, Dmoszynska A, Skomra D, Wasik-Szczepanek E, Szumilo J: **Intravascular B-cell lymphoma in a 38-year-old woman: a case report**. *Annals of hematology* 2001, **80**(4):224-227.
399. Wahie S, Dayala S, Husain A, Summerfield G, Hervey V, Langtry JA: **Cutaneous features of intravascular lymphoma**. *Clinical and experimental dermatology* 2011, **36**(3):288-291.
400. Wake A, Kakinuma A, Mori N, Nagata K, Tsukada J, Nakata K, Misago M, Oda S, Morimoto I, Eto S: **Angiotropic lymphoma of paranasal sinuses with initial symptoms of oculomotor nerve palsy**. *Internal medicine* 1993, **32**(3):237-242.
401. Walker UA, Herbst EW, Ansorge O, Peter HH: **Intravascular lymphoma simulating vasculitis**. *Rheumatology international* 1994, **14**(3):131-133.
402. Walls JG, Hong YG, Cox JE, McCabe KM, O'Brien KE, Allerton JP, Derdak S: **Pulmonary intravascular lymphomatosis: presentation with dyspnea and air trapping**. *Chest* 1999, **115**(4):1207-1210.
403. Wang BY, Strauchen JA, Rabinowitz D, Tillem SM, Unger PD: **Renal cell carcinoma with intravascular lymphomatosis: a case report of unusual collision tumors with review of the literature**. *Archives of pathology & laboratory medicine* 2001, **125**(9):1239-1241.
404. Wang L, Li C, Gao T: **Cutaneous intravascular anaplastic large cell lymphoma**. *Journal of cutaneous pathology* 2011, **38**(2):221-226.
405. Waring WS, Wharton SB, Grant R, McIntyre M: **Angiotropic large B-cell lymphoma with clinical features resembling subacute combined degeneration of the cord**. *Clinical neurology and neurosurgery* 1999, **101**(4):275-279.
406. Watabe R, Shibata K, Hirase N, Kodera T, Muta K, Nishimura J, Nawata H: **Angiotropic B-cell lymphoma with hemophagocytic syndrome associated with syndrome of inappropriate secretion of antidiuretic hormone**. *Annals of hematology* 2000, **79**(10):581-584.
407. Weichert G, Martinka M, Rivers JK: **Intravascular lymphoma presenting as telangectasias: response to rituximab and combination chemotherapy**. *Journal of cutaneous medicine and surgery* 2003, **7**(6):460-463.
408. Weisel KC, Brugger W, Krober SM, Kaiserling E, Kanz L: **Intravascular lymphoma - a rare cause of hemolytic anemia and neurologic disorders**. *The*

hematology journal : the official journal of the European Haematology Association / EHA 2004, **5**(5):444-446.
409. Weitten T, Guiot P, Mootien Y, Rozan-Rodier S, Andres E: **Multiorgan failure caused by intravascular lymphoma**. *QJM : monthly journal of the Association of Physicians* 2008, **101**(6):508-510.
410. Wick MR, Banks PM, McDonald TJ: **Angioendotheliomatosis of the nose with fatal systemic dissemination**. *Cancer* 1981, **48**(11):2510-2517.
411. Wick MR, Scheithauer BW, Okazaki H, Thomas JE: **Cerebral angioendotheliomatosis**. *Archives of pathology & laboratory medicine* 1982, **106**(7):342-346.
412. Willemze R, Kruyswijk MR, De Bruin CD, Meijer CJ, Van Berkel W: **Angiotropic (intravascular) large cell lymphoma of the skin previously classified as malignant angioendotheliomatosis**. *The British journal of dermatology* 1987, **116**(3):393-399.
413. Williams DB, Lyons MK, Yanagihara T, Colgan JP, Banks PM: **Cerebral angiotropic large cell lymphoma (neoplastic angioendotheliosis): therapeutic considerations**. *Journal of the neurological sciences* 1991, **103**(1):16-21.
414. Williams G, Foyle A, White D, Greer W, Burrell S, Couban S: **Intravascular T-cell lymphoma with bowel involvement: case report and literature review**. *American journal of hematology* 2005, **78**(3):207-211.
415. Williams RE, Seywright MM, Lever R, Lucie NP: **Angiotropic B-cell lymphoma (malignant angioendotheliomatosis): failure of systemic chemotherapy**. *The British journal of dermatology* 1990, **123**(6):807-810.
416. Wong P, Moonie A, Dennett X, Anpalahan M: **A case of intravascular lymphomatosis (IVL) presenting as polyneuropathy**. *European journal of internal medicine* 2006, **17**(1):59-60.
417. Wrotnowski U, Mills SE, Cooper PH: **Malignant angioendotheliomatosis. An angiotropic lymphoma?** *American journal of clinical pathology* 1985, **83**(2):244-248.
418. Wu H, Said JW, Ames ED, Chen C, McWhorter V, Chen P, Ghali V, Pinkus GS: **First reported cases of intravascular large cell lymphoma of the NK cell type: clinical, histologic, immunophenotypic, and molecular features**. *American journal of clinical pathology* 2005, **123**(4):603-611.
419. Wu SJ, Chou WC, Ko BS, Tien HF: **Severe pulmonary complications after initial treatment with Rituximab for the Asian-variant of intravascular lymphoma**. *Haematologica* 2007, **92**(1):141-142.
420. Xanthopoulos V, Galanopoulos AG, Paterakis G, Apessou D, Argyrakos T, Goumakou E, Papadhimitriou SI, Savvidou I, Georgiakaki M, Anagnostopoulos NI: **Intravascular B-cell lymphoma with leukemic presentation: case report and literature review**. *European journal of haematology* 2008, **80**(2):177-181.
421. Xia C, Lang SY, Li XM, Yu SY: **Intravascular lymphomatosis with recurrent cerebral hemorrhages**. *Neurology India* 2009, **57**(6):817-819.
422. Xu M, Yang Q, Li M, Geng W, Huang W, Chen Y: **Prostate involvement by intravascular large B-cell lymphoma: a case report with literature review**. *International journal of surgical pathology* 2011, **19**(4):544-547.

423. Yalamanchili M, Prabhu S, Bradstreet P, Chouhdry I, Walsh J: **Intravascular lymphomatosis presenting as systemic inflammatory response syndrome**. *The American journal of the medical sciences* 2002, **324**(6):339-341.
424. Yamada N, Uchida R, Fuchida S, Okano A, Okamoto M, Ochiai N, Iwasa H, Shimazaki C: **CD5+ Epstein-Barr virus-positive intravascular large B-cell lymphoma in the uterus co-existing with huge myoma**. *American journal of hematology* 2005, **78**(3):221-224.
425. Yamada S, Nishii R, Oka S, Higashi T, Yagi M, Satow T, Suzuki T, Saiki M: **FDG-PET a pivotal imaging modality for diagnosis of stroke-onset intravascular lymphoma**. *Archives of neurology* 2010, **67**(3):366-367.
426. Yamagata T, Okamoto Y, Ota K, Katayama N, Tsuda T, Yukawa S: **A case of pulmonary intravascular lymphomatosis diagnosed by thoracoscopic lung biopsy**. *Respiration; international review of thoracic diseases* 2003, **70**(4):414-418.
427. Yamaguchi M, Kimura M, Watanabe Y, Taniguchi M, Masuya M, Kageyama S, Katayama N, Ohno T, Kita K, Shiku H: **Successful autologous peripheral blood stem cell transplantation for relapsed intravascular lymphomatosis**. *Bone marrow transplantation* 2001, **27**(1):89-91.
428. Yamamoto T, Morita K, Iriyama N, Wakui K, Hiroi A, Sawada T, Masuda A, Kobayashi M: **Intravascular large B-cell lymphoma of the uterus: a case with favorable clinical outcome**. *International journal of surgical pathology* 2011, **19**(5):672-676.
429. Yanagihori H, Oyama N, Kawakami Y, Sakuma-Oyama Y, Nakamura K, Iwatsuki K, Kaneko F: **A case of intravascular large B-cell lymphoma with multiple organ involvement**. *The Journal of dermatology* 2003, **30**(12):910-914.
430. Yang T, Tian L, Li Q, Liu Y, Zhang Q, Ma Z, Zhang S, Zhou D: **A case of intravascular B-cell lymphoma presenting as myelopathy and diagnosed post mortem**. *Journal of the neurological sciences* 2008, **272**(1-2):196-198.
431. Yao X, Saad A, Chitambar CR: **Intravascular large B-cell lymphoma, an exclusively small vessel disease? A case report and review of literature**. *Leukemia research* 2010, **34**(10):e275-277.
432. Yasuda H, Ando J, Matsumoto T, Ogura K, Ozaki Y, Aritaka N, Aoki Y, Sugita M, Nomura T, Sekii H *et al*: **Intravascular large B cell lymphoma with hepatic portal vein, splenic vein and mesenteric vein tumour embolism**. *Histopathology* 2010, **57**(4):648-650.
433. Yasuda M, Akiyama N, Miyamoto S, Warabi M, Takahama Y, Kitamura M, Yakushiji F, Kinoshita H: **Primary sellar lymphoma: intravascular large B-cell lymphoma diagnosed as a double cancer and improved with chemotherapy, and literature review of primary parasellar lymphoma**. *Pituitary* 2010, **13**(1):39-47.
434. Yegappan S, Coupland R, Arber DA, Wang N, Miocinovic R, Tubbs RR, Hsi ED: **Angiotropic lymphoma: an immunophenotypically and clinically heterogeneous lymphoma**. *Modern pathology : an official journal of the United States and Canadian Academy of Pathology, Inc* 2001, **14**(11):1147-1156.

435. Yeung CK, Trendell-Smith NL, Mak HKF, Lam CCK, Kwong YL: **'Western' or 'Asian' intravascular large B-cell lymphoma?** *Clinical and experimental dermatology* 2009, **34**(7):e482-e483.
436. Yin W, Li M, Gao Z, Huang F, Da J, Liu C: **Intravascular large B-cell lymphoma with involvement of the abdominal subcutis: a case report and literature review**. *International journal of hematology* 2009, **89**(3):348-351.
437. Yoshikawa S, Kobayashi H, Kanda S, Furihata K, Ueno M, Kaneki T, Kodaira H, Ichikawa N, Koyama S, Saito H: **An Asian variant of intravascular lymphoma diagnosed through splenectomy**. *Internal medicine* 2002, **41**(12):1215-1216.
438. Yousem SA, Colby TV: **Intravascular lymphomatosis presenting in the lung**. *Cancer* 1990, **65**(2):349-353.
439. Zeidman A, Horowitz A, Fradin Z, Cohen A, Wolfson L, Elimelech O: **Fulminant intravascular lymphoma presenting as fever of unknown origin**. *Leukemia & lymphoma* 2004, **45**(8):1691-1693.
440. Zhao XF, Sands AM, Ostrow PT, Halbiger R, Conway JT, Bagg A: **Recurrence of nodal diffuse large B-cell lymphoma as intravascular large B-cell lymphoma: is an intravascular component at initial diagnosis predictive?** *Archives of pathology & laboratory medicine* 2005, **129**(3):391-394.

[247-440]I
